# Non-Covalent Interactions and Helical Packing in Thiophene-Phenylene Copolymers: Tuning Solid-State Ordering and Charge Transport for Organic Field-Effect Transistors

**DOI:** 10.1021/acs.chemmater.5c00631

**Published:** 2025-05-23

**Authors:** Manikanta Makala, Zhuang Xu, Shamil Saiev, Xiaojuan Ni, Sina Sabury, Veaceslav Coropceanu, Jean-Luc Brédas, Ying Diao, John R. Reynolds, Oana D. Jurchescu, Anna M. Österholm

**Affiliations:** † Department of Physics and Center for Functional Materials, 8676 Wake Forest University, Winston-Salem, North Carolina 27109, United States; Department of Chemical and Biomolecular Engineering, Department of Chemistry, 14589 Beckman Institute for Advanced Science and Technology, University of Illinois Urbana−Champaign, 600 S. Mathews Avenue, Urbana, Illinois 61801, United States; § Department of Chemistry and Biochemistry, 8041 The University of Arizona, Tucson, Arizona 85721-0041, United States; ∥ School of Chemistry and Biochemistry, Center for Organic Photonics and Electronics, Georgia Tech Polymer Network, 1372 Georgia Institute of Technology, Atlanta, Georgia 30332, United States; ⊥ School of Materials Science and Engineering, Georgia Institute of Technology, Atlanta, Georgia 30332, United States

## Abstract

In this study, we introduce two thiophene-phenylene-thiophene (TPT) polymers designed to leverage noncovalent intramolecular interactions to regulate main-chain conformation and enhance solid-state ordering. By incorporating unsubstituted thiophene (T) or bithiophene (2T) units, we reveal striking divergence in the thermal, morphological, and optoelectronic properties of the resulting films, facilitated by these noncovalent interactions. Using a combination of computational and experimental approaches, we show that annealing yields remarkably different polymer conformations and, consequently, charge transport properties. TPT-T undergoes a significant structural transformation, adopting a more planar backbone conformation and a highly crystalline, edge-on molecular orientation. In contrast, the introduction of a single additional thiophene unit in TPT-2T leads to a more isotropic molecular orientation with a slight preference for face-on alignment, resulting in a heterogeneous film structure that hinders charge transport despite achieving tighter molecular packing. Remarkably, despite being composed of achiral components, TPT-2T develops chirality upon annealing, indicating the formation of a helical conformation. Organic field-effect transistor measurements reveal that the well-ordered alignment in annealed TPT-T films results in higher charge carrier mobility and a narrower distribution of mobility values than in TPT-2T. These findings provide critical insights into the structure–property relationships of conjugated polymers, offering guidance for optimizing molecular design and processing strategies for high-performance organic electronic materials.

## Graphical abstract

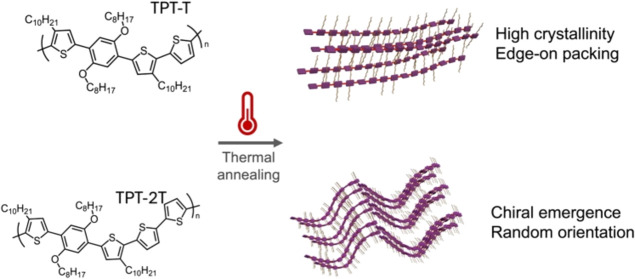

## Introduction

Conjugated polymers (CPs) have emerged as promising materials for various (opto)­electronic and biomedical devices due to their readily tunable optical, electronic, and aggregation properties coupled with amenability for deposition on flexible substrates. This has unveiled possibilities for minimally invasive medical devices and implantable sensors,^1–4^ organic field-effect transistors (OFETs), and organic thermoelectrics, to name a few.^5–16^ The electrical properties of CPs are influenced by polymer conformation and multiscale packing^9,17^ with efficient charge transport typically relying on π-electron delocalization along the polymer backbone and between adjacent chains. It is important to note, however, that high charge carrier mobility has also been observed in near-amorphous polymers, where charge transport is a quasi-one-dimensional, predominantly occurring along the polymer backbone, requiring only occasional intermolecular hopping through short π-stacking bridges.^18–20^ It is generally believed that planarizing the polymer backbone is critical for enhancing charge transport.^21^ Increased planarity and rigidity promote π–π stacking, improving orbital overlap, reducing structural disorder, and lowering charge transport barriers.^21–23^ As a result, carrier mobilities are enhanced and energy landscapes become more uniform. To achieve greater chain planarity, several synthetic strategies have been developed, including the design of conjugated ladder polymers,^15,24^ incorporation of rigid double-bond linkages,^25^ and the use of noncovalent interactions to lock polymer conformation.^26^ Among these, noncovalent locking provides a dynamic approach that maintains solution-state solubility while simultaneously promoting planarity and long-range order in the solid state.^27,28^

Applying noncovalent locking in molecular design can induce a high degree of ordering in the condensed state, resulting in thermotropic liquid crystalline mesophases. We have recently reported that a polymer based on a TPT (1,4-(2-thienyl)-2,5-dialkoxyphenylene) core copolymerized with thienothiophene (TT) units displayed multiple thermotropic liquid crystalline phases upon cooling from its melt state.^28^ Upon processing and subsequent solidification, these interactions become more significant, promoting ordered structures through conformational locking. Side-chain symmetry combined with noncovalent interactions^29^ enables thermal annealing to further enhance this order. As the polymer transitions through nematic, smectic, and additional ordered mesophases, a high degree of crystallinity emerges, as evidenced by well-defined diffraction peaks. Consequently, annealed TPT-TT films exhibit more efficient and uniform transport, i.e., higher charge carrier mobilities and a narrower distribution, in OFETs, compared to as-cast films. These results underscore the role of noncovalent interactions in enhancing solid-state order and device performance. From a synthetic standpoint, noncovalent locking interactions in monomers can restrict free bond rotation around the backbone bonds. In particular, if these noncovalent interactions stabilize specific dihedral angles that lead to deviations from planarity it can result in preferred helical twisting or asymmetry along the growing polymer chain; this can promote axial chirality that then guides the conformation and organization of the polymer chain during synthesis and self-assembly.

In this study, we introduce two polymers based on the TPT core, designed to leverage noncovalent intramolecular S···O, O···H–C and S···H–C interactions to regulate main-chain conformation, with the ultimate goal of enhancing solid-state ordering. These polymers are derived from the previously reported TPT-TT polymer, which is known for forming highly ordered structures postannealing. Incorporating either an unsubstituted thiophene (T) and bithiophene (2T) unit enables a comparative investigation of how these structural differences influence solid-state morphology and charge transport properties. Both polymers undergo multiple phase transitions during postannealing, driven by enhanced ordering facilitated by noncovalent interactions. Interestingly, annealing induces the formation of highly crystalline, out-of-plane structures with well-defined diffraction peaks in TPT-T, whereas TPT-2T adopts a more disordered molecular orientation with fewer diffraction features. Specifically, TPT-2T, while composed of inherently achiral components, exhibits emergent chirality upon annealing, suggesting the formation of a helical polymer conformation. To evaluate the impact of these structural modifications on electrical properties, the polymers were incorporated in OFET devices. We found that annealed TPT-T films had significantly enhanced performance and uniformity compared annealed TPT-2T, demonstrating 5 to 10 times higher charge mobilities with a significantly narrower mobility distribution. Despite exhibiting lower charge carrier mobilities, TPT-2T displayed a significantly lower threshold voltage. Notably, both polymers outperform the TPT-TT analog in terms of OFET characteristics.

## Experimental Section

### Materials

TPT-T and TPT-2T (Figure 1a) were synthesized via Stille cross-coupling (synthetic pathway shown in the Supporting Information, Figure S1) and are of high purity according to ^1^H NMR and elemental analyses (Figures S2–S5). The number-average molecular weights, estimated by size exclusion chromatography, are comparable (TPT-T = 17.2 kg/mol and TPT-2T = 15.7 kg/mol, relative to polystyrene standards) and both polymers have narrow dispersities (*Đ* = 1.6) with monomodal chromatograms (Figure S6). Maintaining comparable molecular weights across the polymer series is critical to ensure that the observed differences in polymer assembly, thermal behavior and transport properties are due to intrinsic factors (i.e., chemical structure) rather than extrinsic factors such as chain length or degree of entanglement.

**Figure 1:**
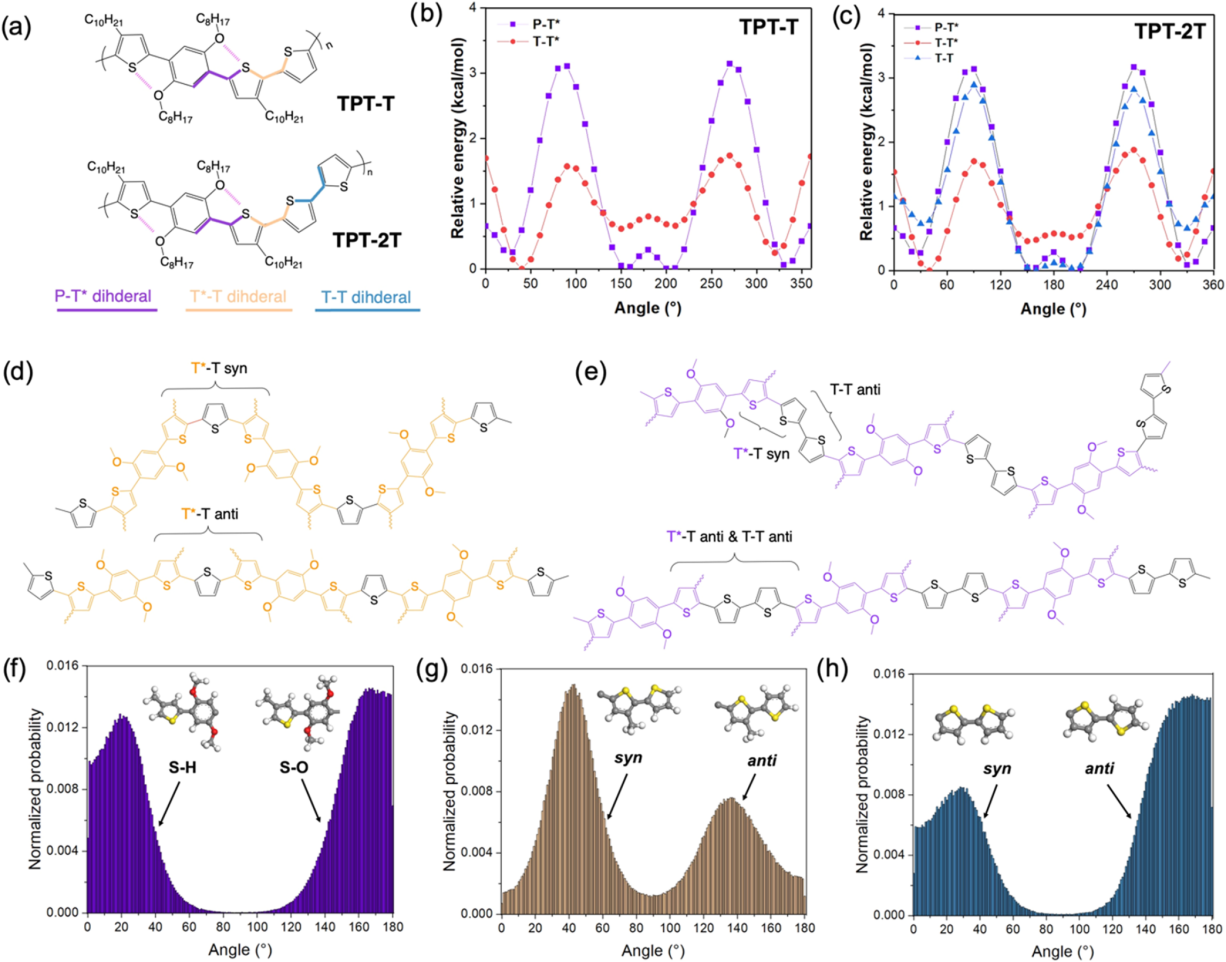
(a) Schematic representation of the backbone dihedrals: phenylene-thienylene (P-T*), thienylene-thienylene (T-T*) and thienylene-thienylene (T-T) in TPT-T and TPT-2T (where an asterisk denotes a thienylene ring within the TPT unit). Torsional potential energies of P-T*, T-T* and T-T dihedrals in (b) TPT-T and (c) TPT-2T repeat units, as calculated at the DFT M06–2*X*/6-31G** level of theory. Schematic representation of the effect of *syn* and *anti*-configurations in (d) TPT-T and (e) TPT-2T, where the top structures incorporate the computationally predicted *syn*-conformations between thienylene (T*) and the thienylene (T) rings and the bottom structures have all thienylene rings in an all-*anti*-conformation. Molecular dynamics simulated torsional populations for (f) P-T, (g) T-T*, and (h) T-T dihedrals in the bulk polymers.

### Quantum-Chemical Calculations and Molecular Dynamics Simulations

Density functional theory (DFT) calculations were performed using the Gaussian 16 package^30^ at the M06-2*X*/6-31G** level of theory. The dihedral angles between the ring units were systematically varied from 0 to 360° in 10° increments, while geometry optimizations were carried out for all other degrees of freedom.

All-atom molecular dynamics (MD) simulations were conducted using the LAMMPS software^31^ and the Optimized Potentials for Liquid Simulations–All Atom (OPLS-AA) force field.^32^ Atom types and force field parameters were generated through the LigParGen web server. Dihedral scans of P-T*, T-T*, and T-T dihedrals (where an asterisk denotes a thienylene ring within a TPT unit) were performed with parameters optimized for the OPLS-AA force field based on DFT calculations. Atomic partial charges were derived by fitting the DFT-calculated electrostatic potential (CM5)^33^ using the same M06-2*X*/6-31G** level of theory. Long-range electrostatics were handled via the particle–particle-particle-mesh (PPPM) method with a root-mean-square accuracy of 10^–5^.^34^ Simulations utilized the Verlet integrator with a 1 fs time step, while temperature and pressure control were maintained using the Nose–Hoover thermostat/barostat. Simulations were carried out under either NVT (constant number of particles, volume, and temperature) or NPT (constant number of particles, pressure, and temperature) ensembles, with a pressure set at 1 atm. A 12 Å cutoff was applied to compute van der Waals interactions.

The bulk structures of TPT-T and TPT-2T polymers were prepared using 60 polymer chains, each with a degree of polymerization of 10. These structures underwent an “annealing” process at 600 K for 25 ns, followed by a gradual cooling to 300 K over an identical period of 20 ns, and subsequently equilibrated for an additional 20 ns. The torsional population was then analyzed on the equilibrated structures. Methyl groups were used as substitutes for the side chains, an approach that effectively bypasses the complications introduced by longer chains, while still considering the significant role played by the side chain bulkiness in determining torsional barriers.

### Film Characterization

#### UV–vis Spectroscopy

Spectroscopic measurements were carried out with an Agilent Cary 5000 spectrophotometer using 1 cm path length quartz cuvettes. For solution UV–vis-NIR spectroscopy measurements the polymers were dissolved in toluene, chloroform, or chlorobenzene at a concentration of 0.1 mg/mL. The thin-film UV–vis absorption spectra were collected on spun coated films from ca. 5 mg/mL chlorobenzene solutions. Temperature-dependent UV–vis spectra were collected to probe the thermochromic behavior of TPT-T and TPT-2T using a Peltier attachment model TC225 from Quantum Northwest. Thin film spectra were recorded on spun coated film on ITO/glass (Delta Technologies, 8–12 ohm/sq).

#### Differential Pulse Voltammetry (DPV) and Cyclic Voltammetry (CV)

DPV and CV were performed on polymer-coated ITO/glass electrodes (active area: 1.4 cm^2^). The electrolyte used for the electrochemical experiments was 0.1 M tetrabutylammonium hexafluorophosphate in acetonitrile (TBAPF_6_/ACN, the salt was recrystallized from hot ethanol). The reference electrode was an Ag/Ag^+^ pseudoreference electrode (inner solution: 10 mM AgNO_3_ in 0.5 M TBAPF_6_ in acetonitrile, calibrated versus Fc/Fc^+^ that had an estimated *E*_1/2_ of 0.140 V). The counter electrode was a Pt flag. Differential pulse and cyclic voltammograms were recorded using an EG&G PAR 273A potentiostat/galvanostat under CorrWare control between −0.5 and 0.8 V vs Ag/Ag^+^. For the DPV measurements, the step size was 2 mV, step time of 0.08 s, and a pulse amplitude of 20 mV. For the CV measurements the scan rate used was 25 mV/s. All experiments were conducted under an argon blanket in degassed electrolyte solutions.

#### Thermal Gravimetric Analysis (TGA) and Differential Scanning Calorimetry (DSC)

Thermogravimetric analysis of TPT-T and TPT-2T were performed on a Mettler-Toledo TGA2 by ramping the temperature from 50 to 800 °C at a rate of 20 °C/min under nitrogen environment. DSC measurements were performed on a DSC 250 from TA Instruments. The thermograms were collected during two heating and cooling cycles from 0 to 250 °C under nitrogen atmosphere, at ramp rate of 10 °C/min.

#### Circular Dichroism (CD)

CD spectra of the films were recorded using a JASCO 1500 spectrophotometer. A nonzero CD ellipticity indicates differential absorption of left- and right-handed circularly polarized light, arising from the chiral assembly of polymers. To eliminate the effects of linear dichroism and birefringence, all samples were examined by averaging four measurements, incorporating 90° in-plane and 180° out-of-plane rotations.^23,35^

#### Cross-Polarized Optical Microscopy (CPOM)

The birefringence of the TPT-T and TPT-2T thin films and powders was characterized using cross-polarized optical microscopy (Nikon Eclipse Ci-POL). A Linkam stage was used to heat/cool the thin film/polymer powder with a speed 10 °C/min. The film/powder was annealed at each recorded temperature for 10 min to ensure reaching the equilibrium state.

#### Grazing-Incidence X-ray Diffraction (GIXD)

GIXD measurements were performed at the 7.3.3 beamline of the Advanced Light Source at Lawrence Berkeley National Laboratory, using incident angles of 0.14° with an X-ray energy of 10 keV, and a beam size of 30 μm × 50 μm. All samples were scanned for 10 s in a helium chamber during the measurement.

### Fabrication and Characterization of OFETs

A staggered bottom-contact/top-gate geometry was employed in the fabrication of OFETs.^36^ Prior to device fabrication, the substrates were cleaned by sequential immersion in hot acetone and isopropanol baths at 85 °C, followed by drying under a nitrogen flow, then UV-ozone treatment, rinsing with deionized water, and a final drying with nitrogen. Source and drain contacts were defined by depositing 40 nm films of Au over 3 nm of Ti onto the substrate through a shadow mask. These substrates were immersed in a solution of 20 μL pentafluorobenzenethiol (PFBT) in 5 mL of anhydrous room-temperature ethanol for 30 min to form the self-assembled monolayer (SAM) over the gold surface. Following this treatment, samples were rinsed with ethanol for 30 s and dried with N_2_ gas. Polymer films were spin-coated in a nitrogen-filled glovebox from a 10 mg/mL solution in room temperature chlorobenzene at 1000 r.p.m for 60 s. Following deposition, one set of films underwent annealing in the same glovebox, without exposure to ambient conditions, on a preheated hot plate, at 250 °C for 10 min, while the other used immediately, as cast. Subsequently, a gate dielectric layer consisting of 1200 nm Cytop (CTL-809-M) was spin-coated over the polymer film at 2000 r.p.m for 60 s. The stack was further annealed on a hot plate under nitrogen at 110 °C for 25 min. All films were then stored in a vacuum desiccator at room temperature for about 12 h. In the final step of fabrication, a 40 nm thick layer of Au was thermally deposited onto the device array through a shadow mask, to create the top gate electrode. The electrical characterization of OFET devices was performed using a Keithley 2614 B source meter controlled by SweepMe! Software (sweep-me.net). All measurements were conducted using a probe station, in the dark under ambient conditions and the mobility was extracted from the saturation regime.^36^

## Results and Discussion

### Effect of Non-Covalent Interactions and Dihedral Energies on TPT-xT Conformations

To investigate the impact of intramolecular interactions on the backbone configuration in the gas phase, we first examined the torsional potentials along the repeat units of both TPT-T and TPT-2T (Figure 1b,c, the optimizations and optimized coefficients for each dihedral are shown in Figure S7 and in Table S1). The calculations reveal that, while the overall torsional profiles in both polymers exhibit similar energy landscapes, key differences emerge in specific dihedral rotations. For the phenylene-thienylene dihedral (P-T*, where T* represents the alkyl-substituted thiophene in the TPT unit), the noncovalent S···O, O···H–C and S···H–C noncovalent interactions are evident and play crucial roles in promoting backbone planarity. The low-energy conformers located between 150–200° are attributed to S···O interactions, while additional stabilization at ∼30 and 330° is associated with O···H–C and S···H–C noncovalent bonds.^37,38^ These interactions are confirmed by calculated short contact distances (2.75 Å for S···O 2.37 Å for O···H–C and 2.71 Å for S···H–C, i.e., shorter than the sum of the van der Waals radii of the involved atoms), which are in good agreement with previously reported values.^37,39^ The stabilization of this dihedral is further supported by additional low-energy conformers at 30 and 330°, attributed to S···CH interactions. These two conformational states display comparable energy levels and are separated by a high-energy *anti*-to-*syn* barrier of Δ*E*_1_ = 3.17 kcal/mol, indicating strong stabilization. In contrast, the rotation of thienylene-thiophene (T*-T, where T refers to the unsubstituted thiophene) is less restricted, with an *anti*-to-*syn* rotational barrier of Δ*E*_2_ = 1.63 kcal/mol, where the *syn*-conformation is notably more stable. The stabilization of the *syn*-conformation results in a more bent or coiled conformation, in contrast to the more linear, rod-like arrangement expected in an all-*anti* configuration (illustrated in Figure 1d,e). The torsional potential of the thienylene-thienylene (T-T) dihedral, present only in TPT-2T, differs from that of T*-T due to the absence of side chains, resulting in a profile more similar to the P-T* dihedral, where the *anti*-conformation is energetically favorable.

MD simulations were carried out with simulation boxes containing 60 polymer chains (degree of polymerization = 10) using an optimized force field. The results, shown in Figure 1f–h, demonstrate a balanced distribution of the P-T* dihedral angles between S···O and S···CH interactions, with a slight preference for S···O interactions in both polymers (Figure 1f). For the T-T* dihedral, both *syn-* and *anti*-local isomers are observed, with the *syn*-population displaying a narrower distribution (full-width at half-maximum (fwhm) = 32°) compared to the *anti*-population (fwhm = 47.24°) (Figure 1g). The dominant dihedral angles (∼40°) are suboptimal for coplanarity. In contrast, the T-T dihedral (Figure 1h), exclusive to TPT-2T, exhibit a more planar angular distribution, akin to the P-T* dihedral, with *anti*-conformations being more populated. These torsional distributions closely align with the potential energy surfaces of the dihedrals shown in Figure 1a,b, highlighting the strong dependence of the bulk conformational behavior on the dihedral energies of the polymer backbone, with long side chains here exerting minimal influence on torsional distribution.

In addition to the segmental planarity driven by intramolecular interactions, factors such as intermolecular π–stacking between rings and chain twisting also influence the backbone configuration. To gain insights into these factors, we performed MD simulations to estimate the radius of gyration (*R*_g_) and π-stacking propensity. Figure S8 shows the temporal evolution of *R*_g_ for TPT-T and TPT-2T, averaged over 60 polymer chains and normalized for the number of rings. It can be observed that TPT-2T exhibits a smaller average *R*_g_ compared to TPT-T. This suggests that the additional thiophene rings introduce more points for potential twisting along the polymer backbone, yielding a more compact structure. MD simulations can also track the evolution of π-stacks in the bulk structure during solidification (simulated here by an annealing process where the system is heated to 600 K for 30 ns and then cooled to 300 K for 30 ns). The π-stacking interaction is defined by calculating the center of mass and normal vector for each conjugated ring, then evaluating pairs based on a distance threshold of 4.5 Å and alignment criteria requiring the angle between their normal vectors to be within 0–30° or 150–180° (Figure S9). As expected, increasing the temperature to 600 K results in a significant decrease in the number of π-stacks, which then increases during cooling until the bulk structure stabilizes. Interestingly, despite the greater backbone twisting predicted for TPT-2T based on *R*_g_ calculations, the π-stacking concentration is nearly double that of TPT-T (0.73 mol/L vs 0.42 mol/L), indicating that the additional thiophene rings enhance interchain π–π interactions by increasing the number of accessible stacking configurations, ultimately facilitating the formation of a densely packed and energetically stable network despite conformational distortions.

UV–vis spectra in dilute solutions (Figure 2a,b) reveal similar solvation behaviors for TPT-T and TPT-2T in toluene, chlorobenzene, and chloroform, with a maximum absorption (λ_max_) at 450 nm. Temperature-dependent UV–vis measurements in toluene (10–80 °C) confirm complete dissolution, with minimal spectral shifts and no signs of aggregation (Figure S10). Upon solidification, both polymers display a red-shifted absorption and the emergence of vibronic peaks indicating extended conjugation and/or enhanced molecular ordering, along with stronger electronic-vibrational coupling in the solid state. The 0–0 (568 nm, 2.18 eV) and 0–1 (528 nm, 2.35 eV) peaks in TPT-T are further red-shifted compared to those in TPT-2T (550 and 520 nm/2.25 and 2.38 eV), suggesting that chain twisting (as estimated by *R*_g_) has a larger influence on backbone conformation than the π-stacking propensity in as-cast TPT-based films. This structural distinction is further supported by ionization potential estimations from differential pulse voltammetry. In the as-cast state, TPT-T has an onset of oxidation that is 0.1 V lower than TPT-2T (5.19 vs 5.30 eV) reflecting somewhat greater electron delocalization (Figure 2c, assuming Fc/Fc^+^ is −5.12 eV vs vacuum, cyclic voltammograms and additional differential pulse voltammograms of as-cast and annealed samples can be found in Figure S11).

**Figure 2:**
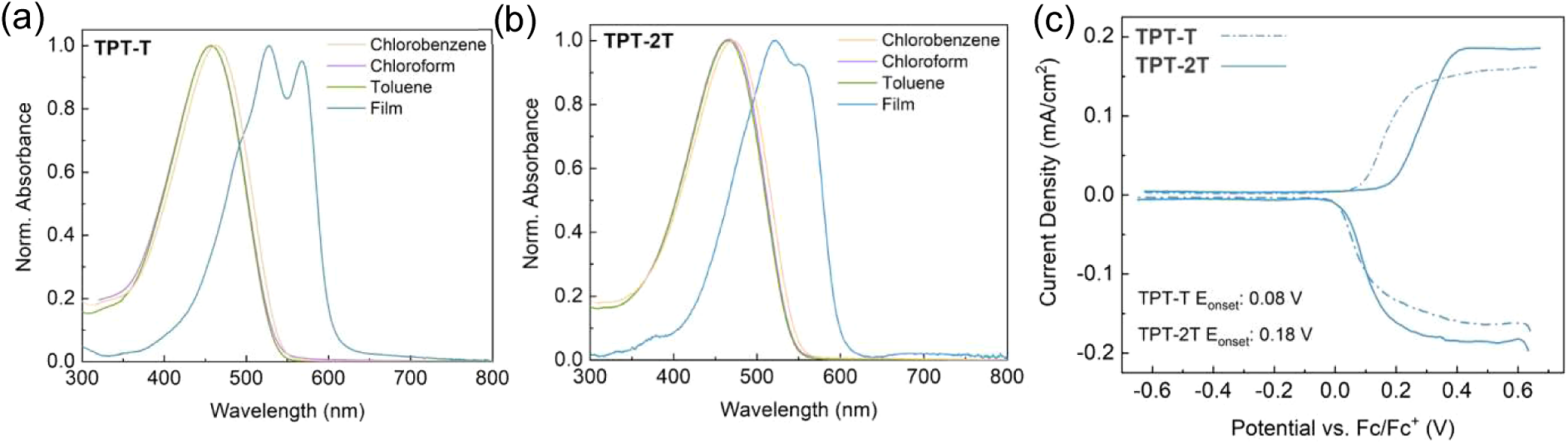
UV–vis spectra of 0.1 mg/mL solutions of (a) TPT–T and (b) TPT-2T in chlorobenzene (orange trace), chloroform (purple trace), and toluene (green trace) at room temperature, as well as their corresponding thin-film spectra (blue trace, spun-coated from 5 mg/mL chlorobenzene solutions). (c) Differential pulse voltammograms of as-cast TPT-T (dashed lines) and TPT-2T (solid lines) films on ITO/glass in 0.1 M TBAPF_6_/ACN. The voltammograms were obtained on films that had not undergone any electrochemical cycling to ensure that redox properties reflect the behavior of as-cast film morphologies.

### Morphology Tuning through Thermal Annealing

Thermal annealing effectively transforms the nonequilibrium morphologies formed during solution coating into more thermodynamically stable structures.^15,40^ This postprocessing treatment is particularly effective for conjugated polymers, especially thermotropic liquid crystalline systems, where heating to the melt state and subsequently cooling in a controlled manner can allow the polymer chains to traverse multiple liquid crystalline mesophases at equilibrium, resulting in significantly improved crystallinity.^28^ Additionally, annealing addresses challenges posed by volatile solvents used during film processing (e.g., chloroform or toluene) by eliminating kinetically trapped film structures, thereby allowing the polymer chains to reorganize into more thermodynamically favored, equilibrium liquid crystalline mesophases.

We first examined the effects of thermal annealing on the morphology of TPT-T and TPT-2T using TGA, DSC, and CPOM. TGA confirmed that the polymers are thermally stable up to 400 °C (Figure S12). The DSC scan of TPT-T powder (Figure 3a) reveals two well-defined endothermic transitions during the second heating cycle at 115 and 157 °C, paired with exothermic transitions at 108 and 136 °C. In contrast, TPT-2T powder (Figure 3b) shows a more complex thermogram, with three broad transitions observed during the second heating cycle at ∼40, 82, and 153 °C, and a sharper peak at 170 °C alongside a crystallization peak at 169 °C during cooling. The negligible temperature difference (ΔT) between the melting and crystallization peaks at 170 and 169 °C, respectively, points to the presence of liquid crystalline mesophases in TPT-2T. In-situ CPOM confirms this, revealing the emergence of liquid crystalline textures and temperature dependent changes in birefringence when a TPT-2T powder is heated above its melting point and then cooled (Figure S13). At 170 °C, a biphasic isotropic–nematic phase is observed, along with the formation of liquid crystalline droplets known as tactoids (Figure S14).^41^ A TPT-T powder, on the other hand, does not display any evidence of thermotropic liquid crystalline phases, as no birefringence is observed under CPOM (Figure S15). Lastly, we assessed the impact of the thermal transitions on the morphology of spin-coated films. CPOM analysis (Figure 3c,d) shows that, after annealing and cooling from the melt state, both films exhibit enhanced crystallinity and molecular alignment, transitioning from nonbirefringent as-spun films to birefringent films under cross-polarized light. Notably, TPT-2T films exhibit distinct birefringent domains with Schlieren textures, confirming its liquid crystallinity whereas TPT-T films exhibit birefringence but lack clear liquid-crystalline textures.

**Figure 3:**
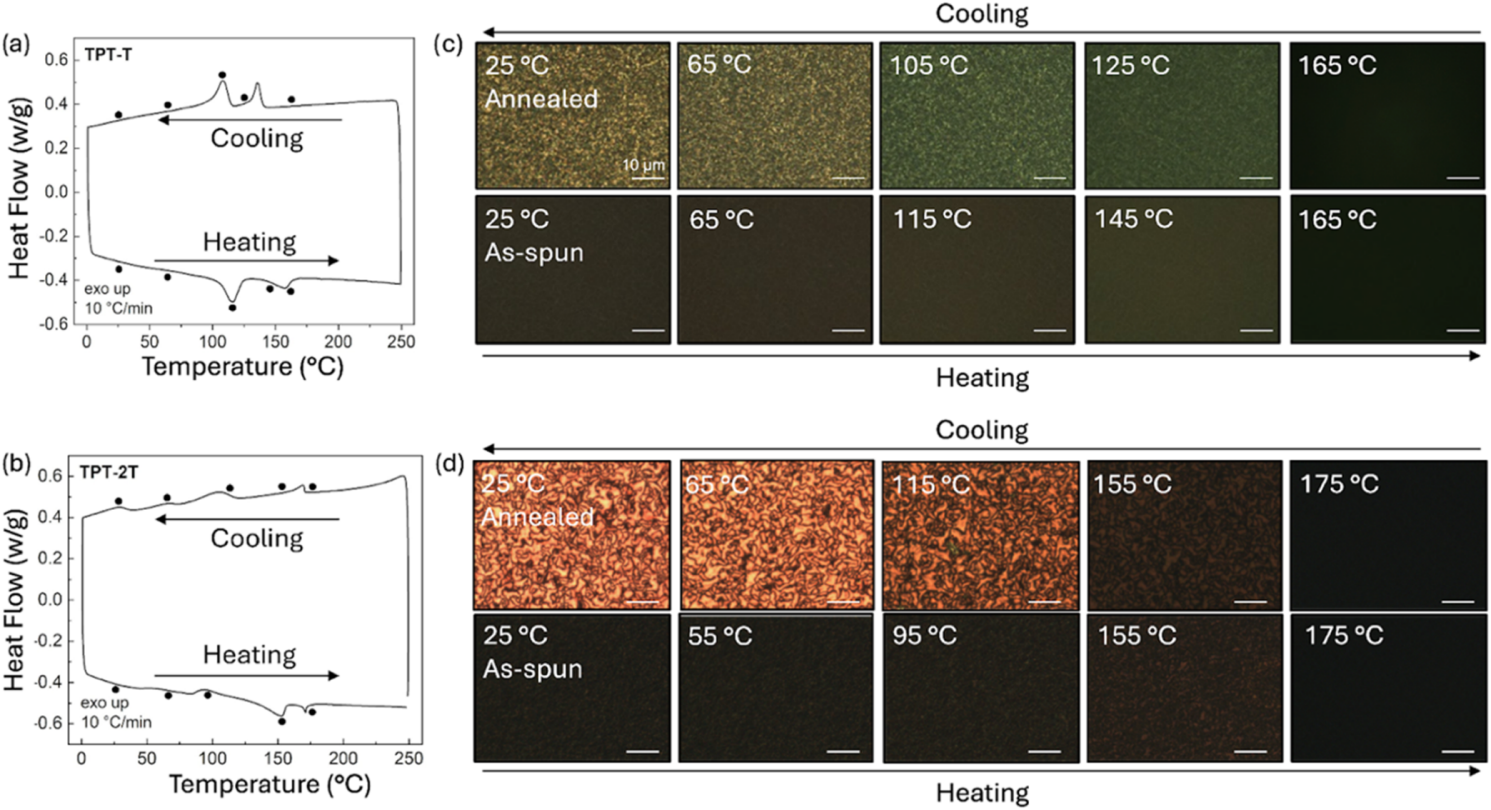
DSC of the first cooling and second heating thermograms of (a) TPT-T and (b) TPT-2T powders. The thermograms were recorded from 0 to 250 °C under nitrogen atmosphere at ramp rate of 10 °C/min. In situ CPOM images of first heating and following cooling of as-spun (c) TPT-T and (d) TPT-2T films. The temperatures at which images were taken correspond to the dot markers on the DSC scans, with the films being equilibrated at each temperature for 10 min.

We further investigated the effects of annealing on the molecular packing, backbone conformation, and mesoscale morphology of TPT-T and TPT-2T using GIXD and UV–vis-NIR. GIXD images (Figure 4a,b) reveal significant changes after film annealing. For TPT-T, multiple sharp diffraction peaks appear, indicating a marked increase in crystallinity. In contrast, the annealed TPT-2T sample shows far fewer diffraction peaks, featuring two broad and tilted π–π stacking peaks centered at azimuthal angles of χ = ± 25° at a *q*_r_ position of 1.58 Å^–1^. These peaks resemble those associated with helical packing along a helical pitch in chiral supramolecular structures.^42^ CD measurements (Figure 4d) confirm this observation by showing nonzero CD ellipticity in TPT-2T, which provides evidence for chiral behavior. In contrast, the TPT-T films remain achiral, as indicated by absence of CD ellipticity (Figure 4c).

**Figure 4:**
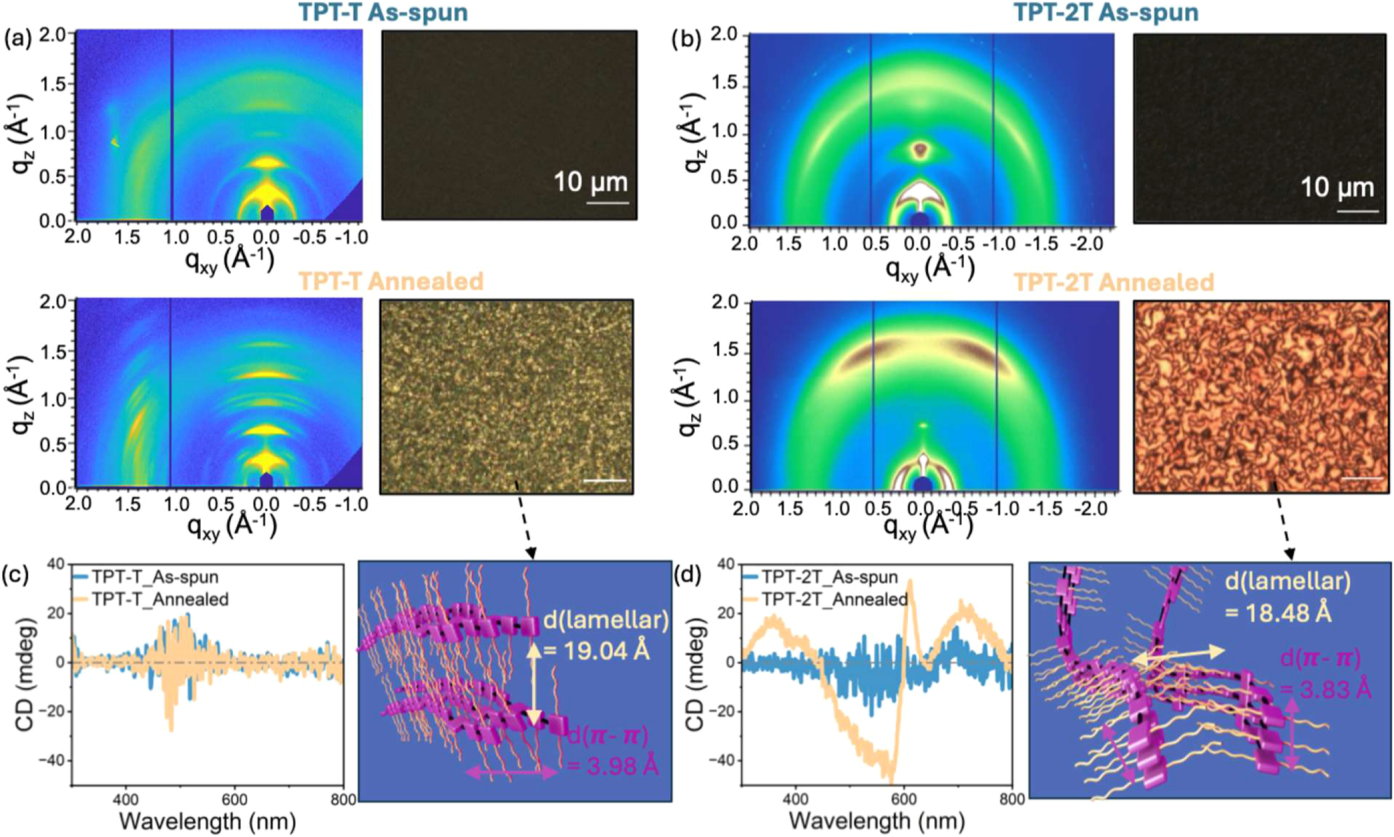
GIXD patterns and corresponding CPOM images of (a) TPT-T and (b) TPT-2T thin films before and after thermal annealing. For TPT-T, thermal annealing results in numerous sharp GIXD peaks and enhanced birefringence under CPOM, indicating increased crystallinity. In TPT-2T films, annealing produces two tilted π–π stacking peaks alongside CD and Schilieren-like textures in CPOM, suggesting chiral liquid crystal-mediated structural ordering. CD spectra and structural illustrations for (c) TPT-T and (d) TPT-2T in as-spun and annealed states. TPT-T shows zero ellipticity, confirming its achiral nature, while TPT-2T exhibits nonzero ellipticity due to the formation of chiral mesophases during annealing.

In addition to providing information on the degree of crystallinity, GIXD scattering patterns also reveal differences in molecular orientation between the two polymers. Analysis of geometrically corrected π–π peak intensity (sin­(χ)­I­(χ)) at different χ provides information on relative population of face-on vs edge-on crystallites.^23,43^ As mentioned above, and as shown in Figure S16, the GIXD patterns for TPT-2T exhibit two prominent π–π stacking peaks at azimuthal angles χ = ± 25° and a *q*_r_ value of 1.58 Å^–1^. A geometry-corrected 1-D intensity plot further shows a slightly face-on, close to isotropic orientation of the π-stacks in the annealed TPT-2T films. In sharp contrast, the TPT-T annealed films display a much more intense π–π stacking peak at χ = 65° (*q*_r_ = 1.58 Å^–1^) compared to χ = 5° (Figure S17), indicating a predominantly near-edge-on molecular orientation. Moreover, TPT-2T exhibits tighter lamellar and π–π stacking spacings compared to TPT-T (Figure 4c,d). UV–vis–NIR spectra before and after annealing (Figure S18) show that in TPT-T, annealing induces a red shift, sharper vibronic structure, and higher absorption coefficient—reflecting enhanced molecular order also on the chromophore length scale. In contrast, despite significant changes in long-range ordering in annealed TPT-2T, its chromophore-scale absorption profile remains essentially unchanged.

From our MD simulations the enhancement in crystallinity, in particular in TPT-T, seem to be the result of the polymer adopting a more rod-like conformation postannealing. Given its more ordered structure, we focused on TPT-T to determine the conformationally plausible crystal structures. This was done by constructing multiple TPT-T conformers and by adjusting the P-T* and T*-T dihedral angles, which results in varying lamellar and π–π stacking patterns. Using the torsional potential energies of the P-T* and T*-T dihedral angles in TPT-T (Figure 1b), two conformers were designed (Conformers A and B, Figure 5a) that feature distinct spatial distributions of side chains. A third conformer was constructed by flipping the T*-T dihedral angle from *syn*-to-*anti*, while a fourth conformer was created by flipping the S–O bond to S–H in the P-T* dihedral angle (Conformers C and D, respectively, in Figure 5a). Of these conformers, Conformer A was found to be the most stable (Figure 5b). While Conformers A and B exhibit similar dihedral angle distributions along the backbone, Conformer A has more ordered side chain spatial distributions, which lowers the energy by 15 meV/monomer compared to conformer B. In contrast, Conformers C and D show significant energy increases due to the disruption of the stable P-T* and T*-T dihedrals, respectively (see Figure 5b). Energetically, this alignment is consistent with the results of the MD simulations presented in Figure 1f,g. We note that Conformer D adopts a less stable P-T* dihedral angle of ∼ 100° due to the steric hindrance from the ordered alkyl side chains, leading to higher energy compared to the other conformers. The optimized structures of all conformers are depicted in Figure 5c and their corresponding lattice parameters are summarized in Table S2. After structural optimization, all conformers exhibited densities exceeding 1.0 g/cm^3^. To further evaluate these proposed crystal structures, we simulated their GIXD images (Figure 5d). Among the four conformers, Conformer A demonstrated the best agreement with experimental GIXD data shown in the bottom panel of Figure 4a. The other three conformers showed shorter packing distances along the lamellar direction, resulting in larger Q_*z*_ values between neighboring rings compared to those observed for Conformer A or in the experimental obtained scattering pattern.

**Figure 5:**
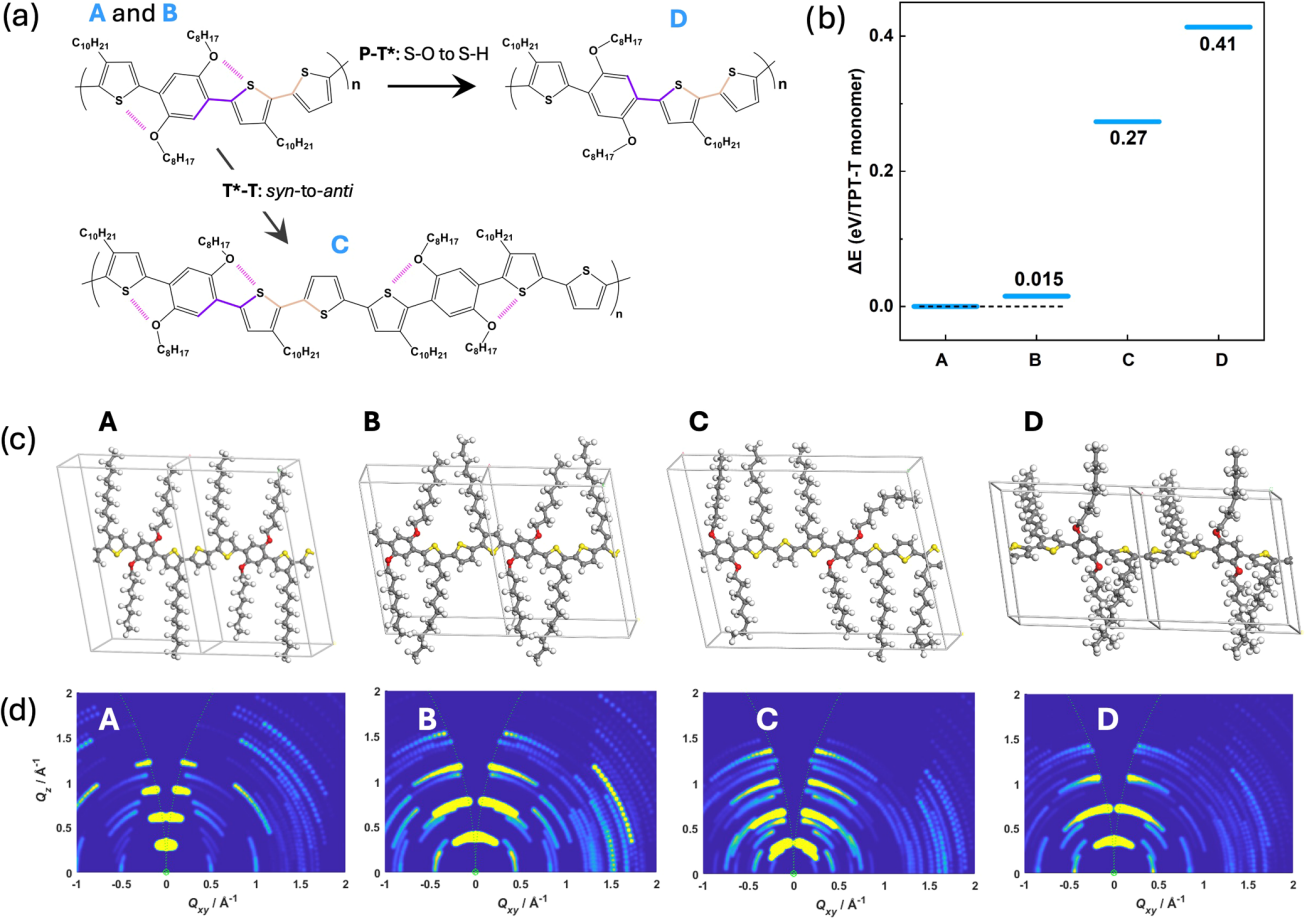
(a) Schematic representations of the backbone dihedrals for four proposed conformers of TPT-T. Conformer A and B share similar backbone dihedrals, while Conformer C was derived by a T*-T *syn*-to-*anti* transformation and Conformer D, by flipping S–O to S–H for P-T* dihedral. (b) The energetics each of the four proposed conformers. (c) Geometrical structures of the four investigated conformers of TPT-T with (d) the corresponding simulated GIXD scattering patterns. The predesigned structures were optimized using DFT calculations within the generalized gradient approximation (GGA) framework, employing the Perdew–Burke–Ernzerhof (PBE) functional as implemented in the Vienna Ab Initio Simulation Package (VASP).^44–46^ The GIXD patterns were simulated using SimDiffraction.^47^ More details can be found in the Supporting Information.

We therefore propose that the higher crystallinity observed in TPT-T, as evidenced by the emergence of multiple GIXD peaks after annealing (Figure 4a,b), likely stems from the lower “energetic cost” associated with adopting a rod-like structure, favored in the crystalline state (Figure 1d,e). Crystal structure calculations (Figures 5) show that Conformer A has *syn* and *anti*-conformations for both T*-T dihedrals. However, the most stable state of the isolated chain features these dihedrals in the *syn* conformation (Figure 1). Transitioning to a rod-like structure therefore requires flipping one dihedral from *syn* to *anti*, a process calculated to have a low energy barrier (∼1.0 kcal/mol). In contrast, in TPT-2T, the presence of an additional (T) ring, and the significant population of the *syn*-conformation in the T-T dihedral, requires flipping the T-T dihedral within each repeat unit, which carries a much higher energy barrier (∼2.9 kcal/mol). Consequently, TPT-2T requires significantly more energy to adopt a linear conformation compared to TPT-T. This difference explains why TPT-T readily crystallizes, whereas TPT-2T tends to adopt helical conformations.

### Charge Transport and OFET Performance

To understand how the different morphologies impact charge transport, we evaluated the electrical properties of the as-cast and annealed polymer films using OFETs, in a top-gate, bottom contact configuration (Figure 6a).^36^ The contacts were chemically treated with a PFBT to reduce the injection barrier between the electrode and the organic semiconductor, thereby minimizing the contact resistance.^5,48–50^ Establishing a low contact resistance is critical for an accurate extraction of the electrical properties of conjugated polymers.^20,51^ The use of a Cytop layer as gate dielectric results in a low electronic trap density at the semiconductor/dielectric interface, while simultaneously encapsulating the polymer film.^52–54^ We fabricated two types of samples for each polymer: one unannealed and one annealed at 250 °C. More than 30 devices with different channel lengths (*L*) and widths (*W*) were measured across multiple substrates for each sample type and the yield was close to 100%. Figure 6b,c displays representative transfer and output *I–V* curves measured on a TPT-T device based on an annealed film, exhibiting a charge carrier mobility of μ = 0.2 cm^2^/(V s). The curves for unannealed TPT-T films are presented in Figure S19 while the corresponding transfer and output characteristics for TPT-2T devices are depicted in Figures S20 and S21, respectively. All samples exhibited hole-transport, and no evidence of electron transport was found under the tested conditions. The voltage range in these graphs is consistent with values commonly employed for OFET devices utilizing Cytop as the gate dielectric layer with thicknesses comparable to those used here (∼1000–1200 nm).^53,55–57^ While these conditions were sufficient for reliable device operation and parameter extraction in our study, we also note that operating voltages could potentially be reduced further, if required for specific applications, by decreasing the dielectric thickness to correspondingly increase the specific gate capacitance.^5,19^

**Figure 6:**
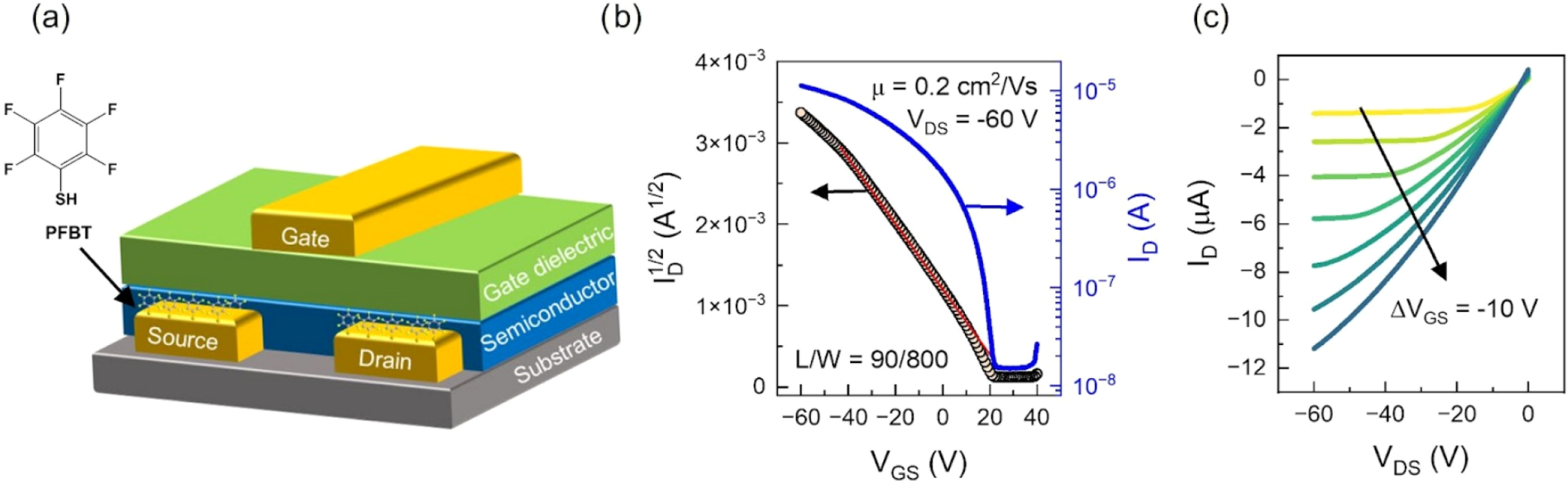
(a) Top-gate, bottom contact OFET device geometry with PFBT SAM-treated contacts. (b) Transfer curve in the saturation regime for an OFET based on annealed TPT-T film. (c) Output characteristics obtained in the same film. The length (L) and width (W) values are 90 and 800 μm, respectively.

A noticeable difference is observed in the output characteristics (Figures 6 and S19), where the annealed devices exhibit less pronounced current saturation compared to the as-cast devices. To investigate the role of contact resistance (*R*_C_) in this behavior, we evaluate its value for the two device types using gated Transmission Line Method (gated-TLM).^36,51^ These measurements revealed that the width-normalized contact resistance (*R*_C_W) remained largely constant before and after thermal treatment, with values of approximately 52.8 kΩ·cm for as-cast films and 51.3 kΩ·cm for annealed films (detailed TLM analysis in Figure S22). While the annealing process enhances charge carrier mobility, and thus lowers the channel resistance (*R*_ch_), the persistence of a significant, unchanged *R*_C_ leads to an increased ratio of contact resistance to channel resistance (*R*_C_/*R*_ch_) in the annealed devices. This increased relative contribution of the contact resistance explains the suppression of saturation observed, particularly at high gate voltages, where the overall device current becomes increasingly limited by the contacts rather than channel pinch-off.

Figure 7 illustrates the mobility histograms for both as-cast and annealed films of TPT-T and TPT-2T, with a summary of all device parameters provided in Table 1. The annealing temperature (250 °C) was intentionally chosen to exceed the temperatures of the observed phase transitions in DSC to ensure complete transformation of the films into the desired phases. Note the narrower distribution in the TPT-T polymer, a feature we will discuss in more detail later. Figure S23 demonstrates no dependence of mobility on geometrical parameters, confirming that the contact resistance is sufficiently low to ensure the extracted mobility values accurately reflect the intrinsic properties of the two polymers.^51^ TPT-T exhibits roughly an order of magnitude increase in mobility after annealing, from an average of μ_avg_ = 1.8 ·10^–2^ cm^2^/(V s) in pristine films to μ_avg_ = 1.5 ·10^–1^ cm^2^/(V s) in annealed films. The maximum mobility values follow the same trend. Along with the charge carrier mobility, the subthreshold slope (SS) represents a critical parameter in OFET operation. SS quantifies the gate voltage required to increase the drain current by an order of magnitude and a lower SS enables faster switching between on and off states with reduced power consumption, making it a highly desirable characteristic. While the SS is slightly reduced from approximately 8 to 5 V/dec upon annealing in our devices, it remains relatively high. The high positive threshold voltages (*V*_th_) observed in TPT-T devices could be attributed to several factors, including the presence of acceptor-like interface states at the semiconductor/dielectric interface, or/and unintentional p-type doping within the semiconductor bulk.^58–62^ We investigated the potential role of atmospheric oxygen as a dopant by comparing device characteristics measured in vacuum (10^–6^ Torr) and ambient air (See Figure S24). We found that all OFET parameters remained consistent across these environments, indicating that ambient oxygen doping or moisture-induced charge scattering is not the dominant factor responsible for the positive *V*_th_ values observed here.^63^ Therefore, the differences in *V*_th_ between our materials likely arise from variations in interface state density or the presence of trace impurities. Compared to TPT-2T, TPT-T devices consistently exhibit a significantly higher positive *V*_th_, suggesting a relatively larger density of interface states or a higher level of residual impurities (Table 1). However, both NMR and elemental analysis results point to the polymers having a high level (and minimal difference) of purity. Furthermore, despite modifying various device parameters and fabrication conditions, including altering solvent purity (grade), measurement environment, and the capacitance of gate dielectric, the *V*_th_ did not decrease significantly. Conversely, TPT-2T shows only a slightly positive *V*_th_ which shifts closer to zero upon annealing. This behavior indicates a comparatively cleaner interface or lower impurity level, with the annealing process likely further reducing interface traps or passivating/removing minor residual impurities, leading to device characteristics closer to ideal enhancement-mode operation.

**Figure 7:**
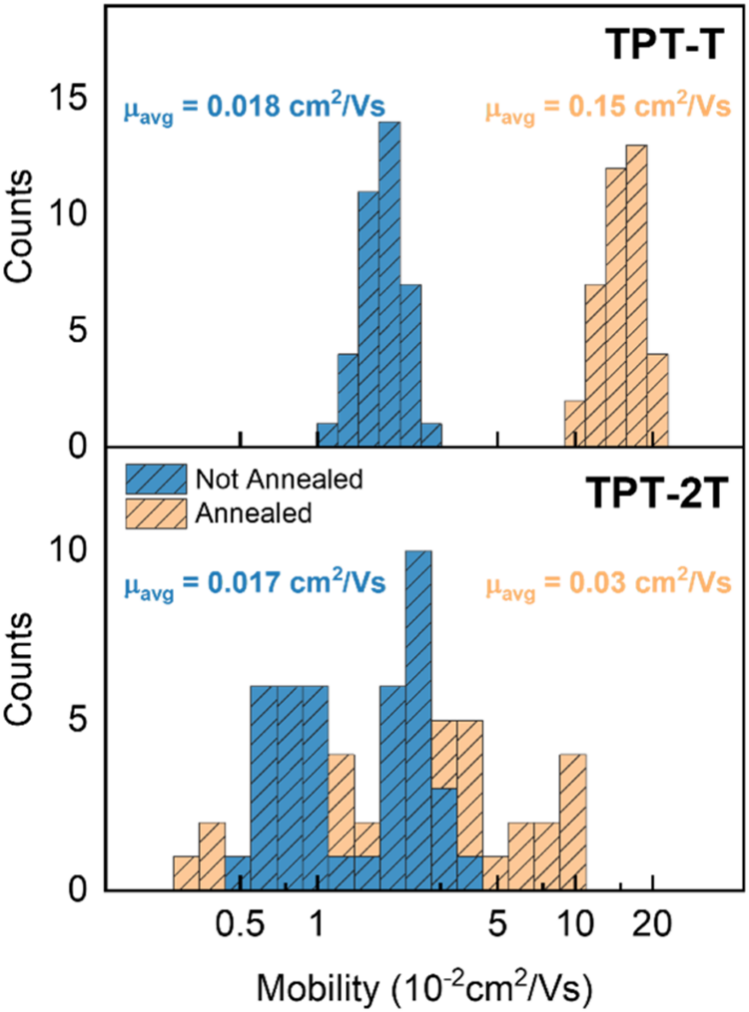
Mobility histograms of OFETs based on TPT-T (top) and TPT-2T (bottom) as cast and annealed films. The average mobility value is included in the inset.

**Table 1:** Summary of OFET Device Parameters

material	annealing	μ_max_ (cm^2^/(V s))	*V*_th_ (V)	on–off	SS (V/dec)	μ_avg_ (cm^2^/(V s))
**TPT-T**	not annealed	0.03	21	10^3^	8.1	(1.8 ± 0.4) × 10^–2^
	annealed	0.2	35	10^3^	5.2	(15 ± 3) × 10^–2^
**TPT-2T**	not annealed	0.04	11	10^6^	1.5	(1.7 ± 0.9) × 10^–2^
	annealed	0.1	0.4	10^6^	1.4	(3.3 ± 2.9) × 10^–2^

The response to annealing in the TPT-2T films is significantly different. Although both maximum and average mobility increase by about a factor of 2 (μ_avg_ = 1.7 ·10^–2^ cm^2^/(V s) in pristine films to μ_avg_ = 3.3 ·10^–2^ cm^2^/(V s) in annealed films), this change falls within the range of sample-to-sample variation and is therefore considered negligible. The SS is notably lower than in TPT-T (approximately 1.5 V/dec) but remains largely unchanged after annealing. On the other hand, the *V*_th_ was reduced to nearly zero postannealing.  This low threshold voltage, combined with the small SS, indicates that the annealed film has achieved a defect-tolerant state.

To understand the contrasting impact of annealing on TPT-T and TPT-2T, we refer to the structural changes induced by this process, drawing upon the computational and experimental results presented earlier. TPT-T undergoes a remarkable transformation upon annealing, adopting a more planar backbone conformation and a highly crystalline structure. Crucially, the molecules predominantly orient in an edge-on configuration relative to the substrate. This preferential alignment facilitates efficient charge transport, explaining the observed increase in mobility and the corresponding narrower distribution of values. The uniformity in orientation ensures consistent pathways for charge carriers, leading to less variation in their mobility. In contrast, TPT-2T exhibits a slightly face-on, close to isotropic molecular orientation after annealing, which includes high-mobility orientations along with a variety of other orientations, resulting in a less efficient charge transport in spite of the tighter molecular packing. The overall film displays a broad distribution of mobility values due to this structural heterogeneity. Furthermore, annealing induces emergent chirality in TPT-2T, leading to the formation of a helical polymer conformation. This helical twisting hinders the conjugation along the polymer backbone, which negatively impacts charge transport. Despite the potential for enhanced mobility due to tighter lamellar and π–π stacking, the detrimental effects of the isotropic orientation and helical conformation ultimately dominate in TPT-2T, preventing efficient transport.

## Conclusions

In summary, we introduced and comparatively analyzed two polymers based on the TPT core, where we leveraged noncovalent intramolecular S···O and S···H–C interactions to control main-chain conformation and ultimately tailor solid-state ordering and charge transport properties. By incorporating unsubstituted thiophene (T) or bithiophene (2T) units, we revealed striking divergence in the thermal, morphological and optoelectronic properties of the resulting films, facilitated by these noncovalent interactions. Our findings demonstrate that annealing has profound impact in TPT-T, leading to a more planar backbone, highly crystalline structure with a predominantly edge-on molecular orientation, which is crucial for efficient interchain charge transport. This favorable morphology translated directly into a remarkable order of magnitude enhancement in OFET mobility and a narrower distribution of mobility values. In contrast, the presence of an additional thiophene unit in TPT-2T resulted in emergent chirality and a helical conformation upon annealing, leading to more isotropic molecular orientation. Consequently, while TPT-2T exhibited a lower threshold voltage, its charge carrier mobility only showed a marginal 2-fold increase and a broad distribution, highlighting the detrimental effects of structural heterogeneity and helical twisting on charge transport efficiency. These conformational differences resulting upon annealing are attributed to the different energy barriers for adopting a rod-like structure. TPT-T requires overcoming a relatively low energy barrier (∼0.5 kcal/mol) for a single dihedral flip from *syn* to *anti*. TPT-2T, on the other hand, with its additional (T) ring and significant population of *syn*-conformation in the T-T dihedral, faces a significantly higher energy barrier (∼2.5 kcal/mol) due to the need for multiple dihedral flips within each repeat unit, favoring the formation of helical structures. This study underscores the intricate interplay between molecular design, noncovalent interactions, and thermal processing in dictating solid-state ordering and charge transport in conjugated polymers. The distinct behaviors of TPT-T and TPT-2T provide valuable insights for the rational design of next-generation polymer semiconductors with tunable electronic properties by manipulating backbone conformation and intermolecular packing through strategic incorporation of specific structural units and controlled processing conditions.

## Supplementary Material



## Notes

Detailed synthesis procedures; molecular dynamic simulations; ^1^H NMR; Size exclusion chromatograms; UV–vis absorption spectra; CPOM imaging; GIXD images; and electrical characteristics of thin film devices (DOCX)
