# Supplementary material for: Non-Covalent Interactions and Helical Packing in Thiophene-Phenylene Copolymers: Tuning Solid-State Ordering and Charge Transport for Organic Field-Effect Transistors
Source: Chem Mater. 2025 May 23;37(11):4145–57. doi: 10.1021/acs.chemmater.5c00631 (PMC12225024; doi:10.1021/acs.chemmater.5c00631)
Supplement: Supplementary file 1 [file cm5c00631_si_001.docx]

**Supporting Information**

**Non-Covalent Interactions and Helical Packing in Thiophene-Phenylene Copolymers: Tuning Solid-State Ordering and Charge Transport for Organic Field-Effect Transistors**

*Manikanta Makala^1,#^, Zhuang Xu^2,#^, Shamil Saiev^3,#^, Xiaojuan Ni^3^, Sina Sabury^4^, Veaceslav Coropceanu^3^, Jean-Luc Brédas^3^, Ying Diao^2^, John R. Reynolds^4,5^, Oana D. Jurchescu^1^, Anna M. Österholm^4,*^*

**Affiliations**:

^1^ Department of Physics and Center for Functional Materials, Wake Forest University, Winston-Salem, NC 27109 United States

^2^ Department of Chemical and Biomolecular Engineering, Department of Chemistry, Beckman Institute for Advanced Science and Technology, University of Illinois Urbana−Champaign, 600 S. Mathews Avenue, Urbana, Illinois 61801, United States

^3^ Department of Chemistry and Biochemistry, The University of Arizona, Tucson, Arizona 85721-0041, United States

^4^ School of Chemistry and Biochemistry, Center for Organic Photonics and Electronics, Georgia Tech Polymer Network, Georgia Institute of Technology, Atlanta, Georgia 30332, United States

^5^ School of Materials Science and Engineering, Georgia Institute of Technology, Atlanta, Georgia 30332, United States


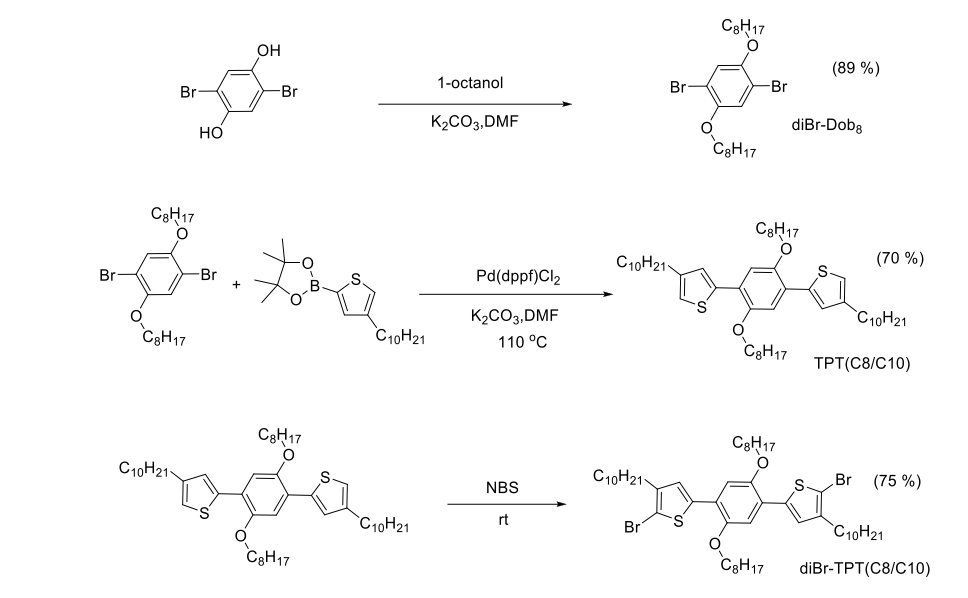


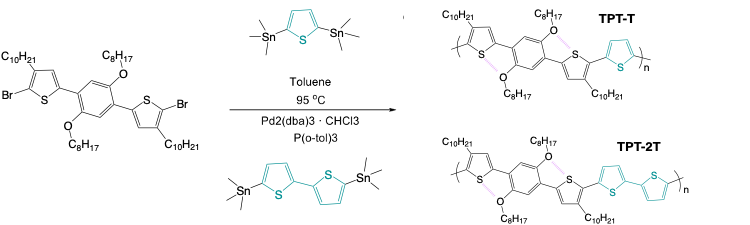


**Figure S1.** Synthetic pathway for TPT-T and TPT-2T. Additional synthetic details and structural characterizations for the TPT core molecule diBr-TPT(C8/C10)^1^.

**Polymerization of TPT-T and TPT-2T**

2,5-bis(trimethylstannyl)thiophene (176 mg, 0.43 mmol, 1 equiv.) or 5,5'-bis(trimethylstannyl)-2,2'-bithiophene (211 mg, 0.43 mmol, 1 equiv.) and dibromo TPT monomer (403 mg, 0.43 mmol, 1 equiv., see **Figures S2-S3** for ^1^H NMR and ^13^C NMR spectra) were added to a freshly dried and cooled round bottom flask equipped with a stir bar. Subsequently, the flask was transferred to a glove box where dipalladium-tris(dibenzylideneacetone)chloroform complex (Pd_2_(dba)_3_·CHCl_3_) was added as the catalyst (21 mg, 0.021 mmol, 0.05 equiv.), along with the ligand tris(o-tolyl)phosphine (26 mg, 0.086 mmol, 0.2 equiv.), and 4 mL of toluene as the polymerization solvent. The reaction flask was then removed from the glove box and immersed in an oil bath set at 105 °C and allowed to stir for 16 hours at this temperature. After this time, a small amount of palladium scavenger (diethylammonium diethyldithiocarbamate) was added, and the temperature was lowered to 90 °C. The solution was then stirred for an additional hour, followed by cooling to room temperature. The crude polymers were precipitated into stirring cold methanol. Purification of the crude polymers was done through Soxhlet washing with methanol (24 hours), acetone (24 hours), and hexane (24 hours). The purified polymers were obtained via Soxhlet extraction using chloroform. Subsequently, the purified polymers were reprecipitated into cold methanol and collected as a red solid by vacuum filtration on a nylon membrane with a pore size of 45 μm. The number-average molecular weight (M_n_) and dispersity (Đ) were determined via gel permeation chromatography using a high-temperature Tosoh EcoSEC equipped with two 7.8 mm x 30 cm, 13 μm TSK-Gel GMHHR-H(S) HT2 columns in series. 1,2,4-trichlorobenzene (TCB) was used as the eluent with a flow rate of 1 mL/min and at a temperature of 140 °C. The instrument was calibrated using polystyrene standards (1,390-1,214,000 g/mol) and the data were analyzed using the 8321GPC-WS analysis software. The polymer samples used for the molecular weight determinations were prepared by dissolving the polymers in TCB at a 1 mg/mL concentration and stirred at 120 °C for at least 3 hours before filtering through a 0.45 µm PTFE filter. The M_n_ and Đ for TPT-T were 17.2 kg/mol and 1.6, respectively. The M_n_ for TPT-2T was 15.7 kg/mol with a Đ of 1.6. Both polymers exhibited monomodal molecular weight distributions as measured by size exclusion chromatography (**Figure S6**). The polymer structure and purity were determined by ^1^H NMR (all monomers and molecular precursors were also characterized by ^13^C NMR using a Bruker Avance IIIHD 500 MHz instruments with CDCl_3_ as the solvent.^1^ Elemental analyses were conducted by Atlantic Microlab Inc.

**^1^H NMR for dibromo TPT** (700 MHz, CDCl3), δ(ppm): 7.20 (s, 1H), 7.12 (s, 1H), 4.07 (t, *J* = 6.5 Hz, 2H), 2.60 (t, *J* = 7.7 Hz, 2H), 1.93-1.88 (m, 2H), 1.71-1.63 (m, 2H), 1.57-1.52 (m, 2H), 1.39-1.24 (m, 22H), 0.91-0.86 (m, 6H) (see **Figure S2)**.

**^13^C NMR for dibromo TPT** (126 MHz, CDCl3), δ (ppm): 149.25, 141.63, 138.44, 125.61, 122.57, 111.44, 110.17, 69.91, 32.07, 32.00, 30.04, 29.81, 29.79, 29.77, 29.64, 29.57, 29.54, 29.51, 29.50, 29.43, 26.46, 22.85, 14.28 (see **Figure S3**).

**^1^H NMR for TPT-T** (500 MHz, CDCl_3_), δ(ppm): 7.45 (s, 2H), 7.25 (s, 2H), 7.15 (s, 2H), 4.15 (t, 4H), 2.86 (t, 4H), 1.97 (quint, 4H), 1.75 (quint, 4H), 1.63 (quint, 4H), 1.49-1.40 (m, 8H), 1.40-1.35 (m, 8H), 1.35-1.23 (m, 28H), 0.89 (m, 12H) (see **Figure S4**).

**Elemental analysis**: Anal. calcd. for C54H84O2S3: C (75.47%), S (11.19%), H (9.62%), O (3.72%); Found: C (74.77%), S (10.94%), H (9.58%).

**^1^H NMR for TPT-2T** (500 MHz, CDCl_3_), δ(ppm): 7.45 (s, 2H), 7.25 (s, 2H), 7.18 (d, 2H), 7.10 (d, 2H), 4.15 (t, 4H), 2.85 (t, 4H), 1.97 (quint, 4H), 1.75 (quint, 4H), 1.63 (quint, 4H), 1.49-1.40 (m, 8H), 1.40-1.35 (m, 8H), 1.35-1.23 (m, 28H), 0.89 (m, 12H) (see **Figure S5**).


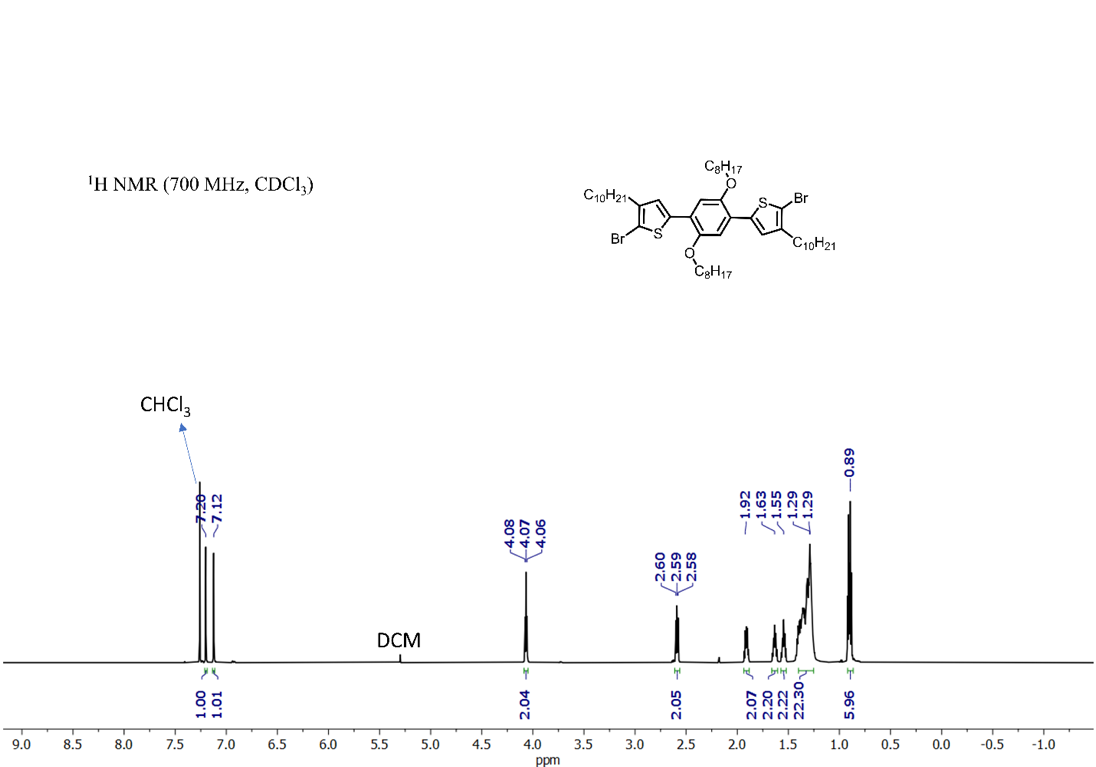
**Elemental analysis:** Anal. calcd. for C58H86O2S4: C (73.99%), S (13.62%), H (8.99%), O (3.40%); Found: C (73.03%), S (13.47%), H (8.87%).


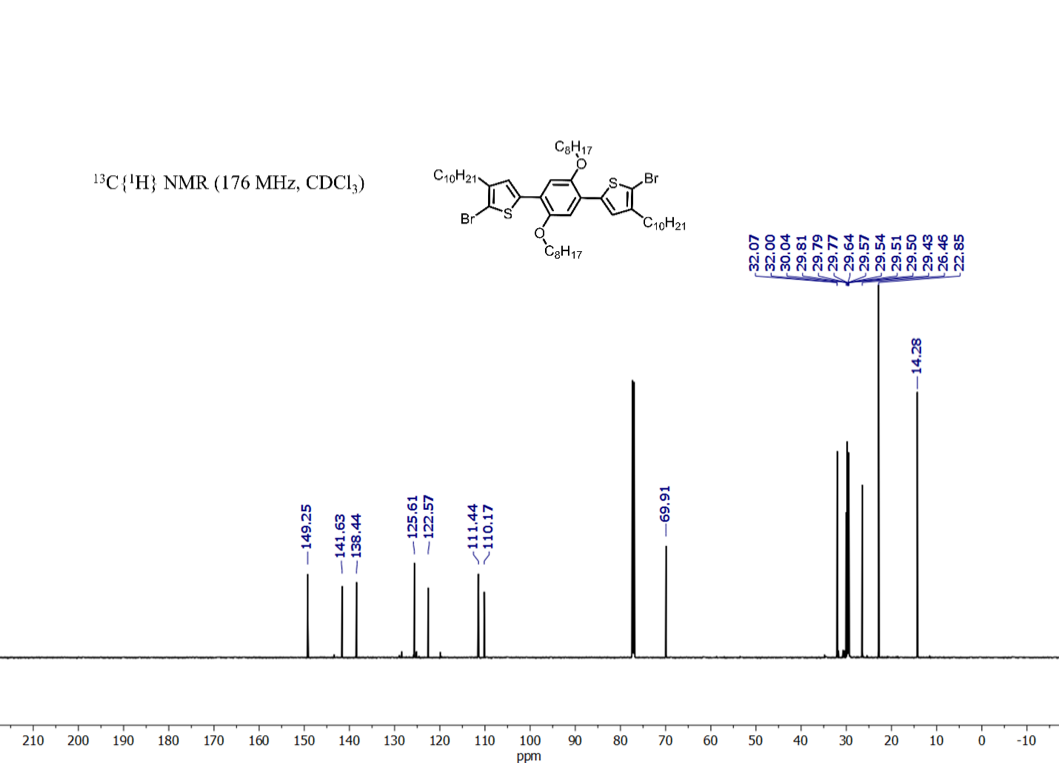
**Figure S2.** ^1^H NMR of dibromo TPT comonomer, 5,5'-(2,5-bis(octyloxy)-1,4-phenylene)bis(2-bromo-3-decylthiophene).

**Figure S3.** ^13^C NMR of dibromo TPT comonomer, 5,5'-(2,5-bis(octyloxy)-1,4-phenylene)bis(2-bromo-3-decylthiophene).


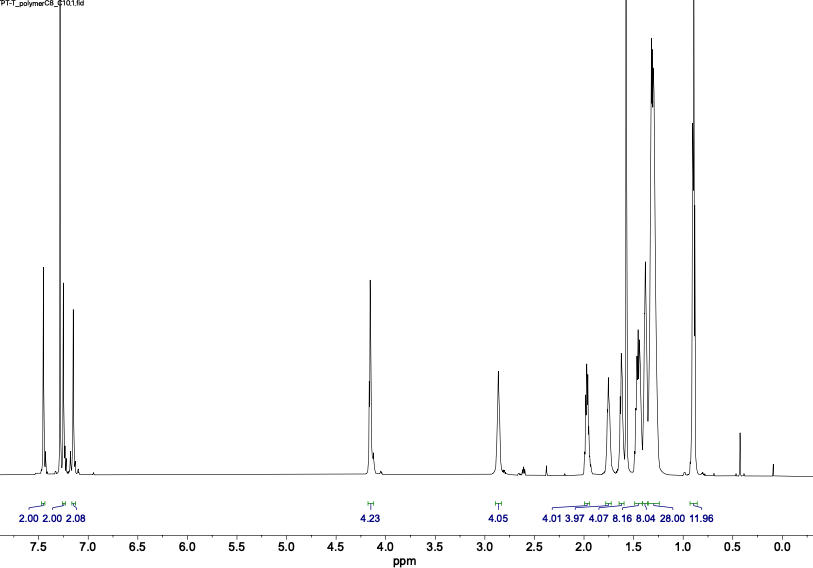


**Figure S4.** ^1^H NMR (500 MHz) of TPT-T in CDCl_3_ measured at room temperature. Residual solvent peaks at 7.26 ppm (CHCl_3_) and 1.56 ppm (H_2_O).


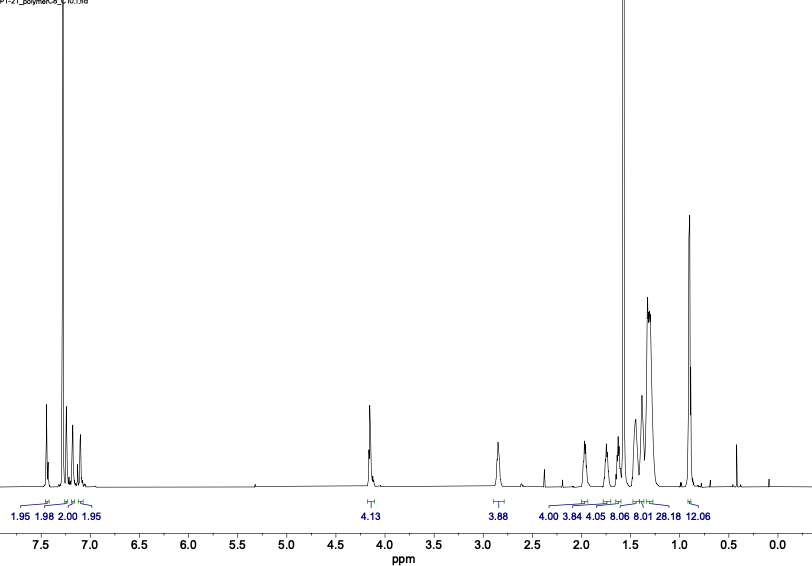


**Figure S5.** ^1^H NMR (500 MHz) of TPT-2T in CDCl_3_ measured at room temperature. Residual solvent peaks at 7.26 ppm (CHCl_3_) and 1.56 ppm (H_2_O).


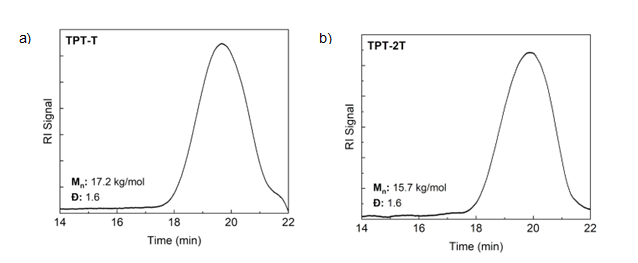
**Figure S6.** Size exclusion chromatograms of a) TPT-T and b) TPT-2T in trichlorobenzene at 140 °C (calibrated vs. polystyrene). The average molecular (M_n_) weight was determined to be 17.2 kg/mol with a Đ of 1.6 for TPT-T and 15.7 kg/mol with a Đ of 1.6 for TPT-2T.

**Table S1.** OPLS-AA optimized force field parameters for the P-T* T-T* and T-T dihedrals (that each include 7 parameters).

|  | **P - T*** | **T - T*** | **T - T** |
| --- | --- | --- | --- |
| **A** | 3.34771 | 1.98277 | 3.1148 |
| **B** | 0.03378 | 0.11734 | 0.3223 |
| **C** | -9.44853 | -7.43088 | -8.63026 |
| **D** | 1.20796 | -0.4343 | -0.01179 |
| **E** | 0.06762 | 1.99882 | 5.29226 |
| **F** | -0.09907 | 0.62207 | 0.22077 |
| **G** | -2.21861 | -1.95376 | -1.84696 |

a)


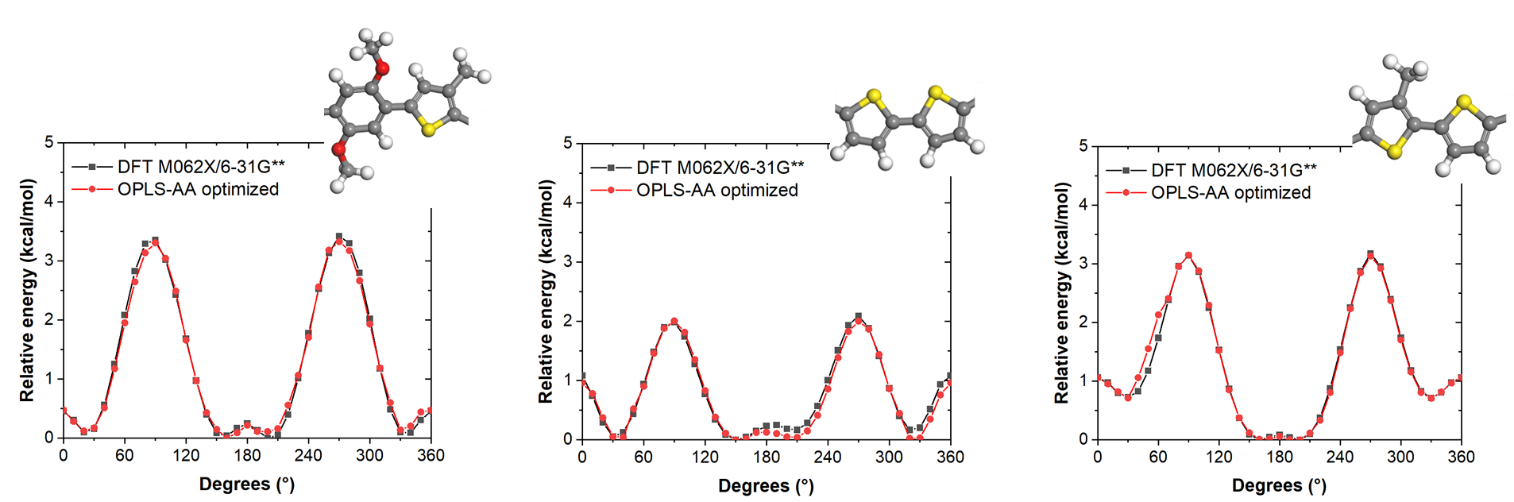


c)

b)

**Figure S7.** Torsional potential energies for the a) P-T*, b) T-T* and c) T-T dihedrals in TPT-T and TPT-2T repeat units, using optimized OPLS-AA force field and comparison with DFT results.


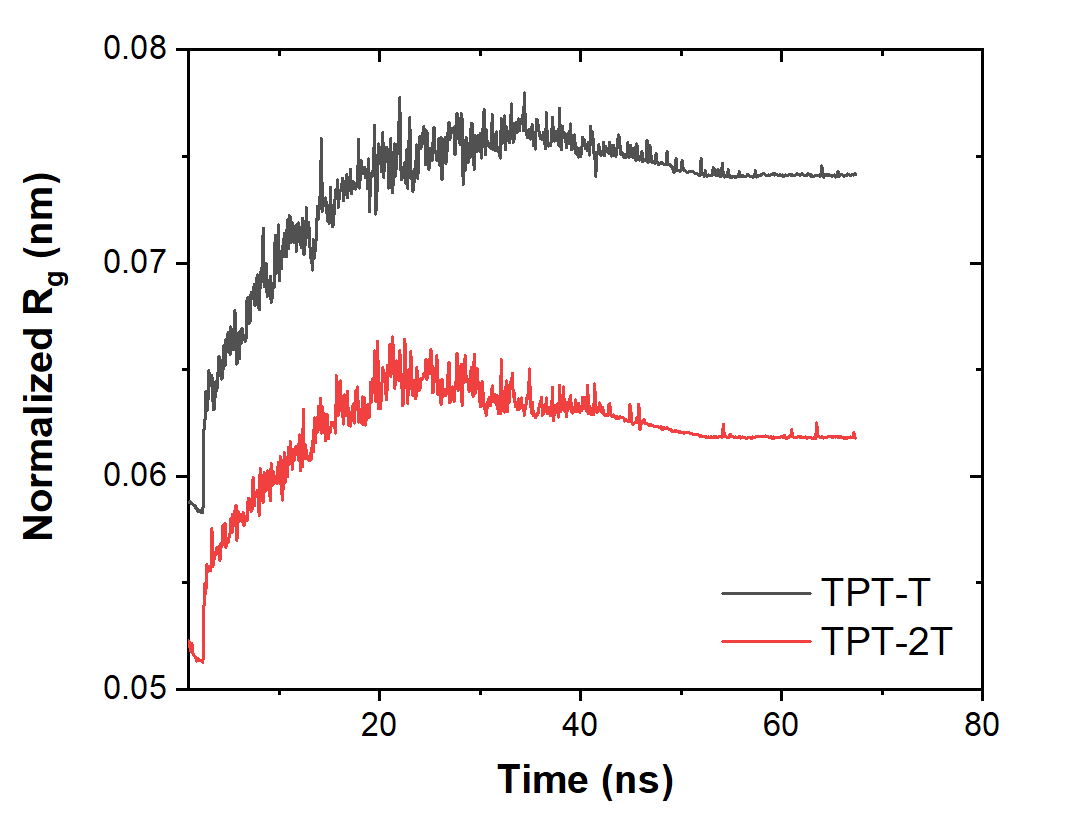


**Figure S8.** Radius of gyration (R_g_) normalized by the number of rings for TPT-T and TPT-2T chains in the bulk during MD annealing of 60 chains. Here, annealing refers to heating the system to 600 K for 30 ns, followed by a gradual cooling to 300 K over an identical period of 30 ns.


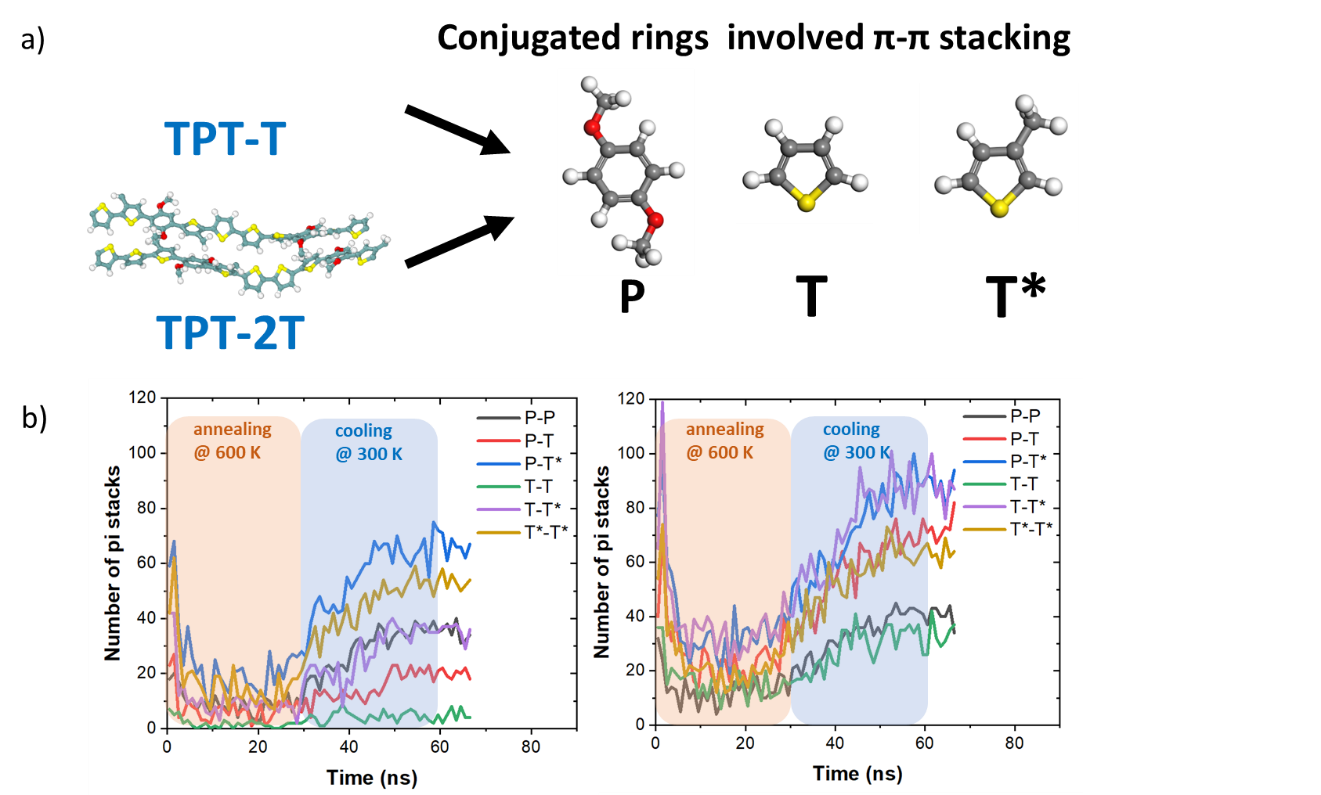


**Figure S9.** a) Fragments of stacked TPT-T and TPT-2T polymer chains and illustration of the conjugated rings involved in π-stacking. Evolution of the number of different π-stack combinations (P-P, P-T, P-T*, T-T, T-T*, and T*-T*) as a function of time during the annealing process for b) TPT-T and c) TPT-2T bulk systems.


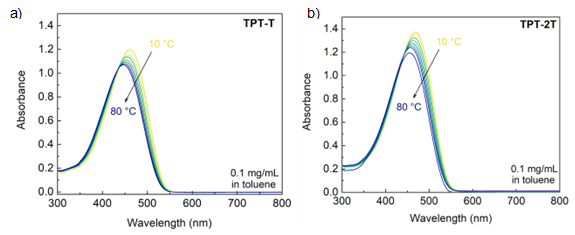


**Figure S10**. Temperature-dependent UV-vis spectra collected 10 °C increments from 10–80 °C of a) TPT-T and b) TPT-2T at 0.1 mg/mL showing negligible evidence of temperature dependent solution aggregation in toluene.


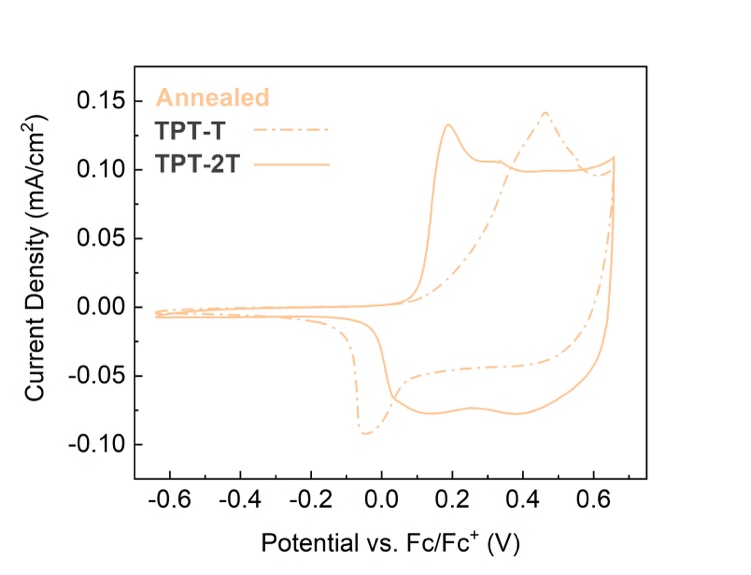

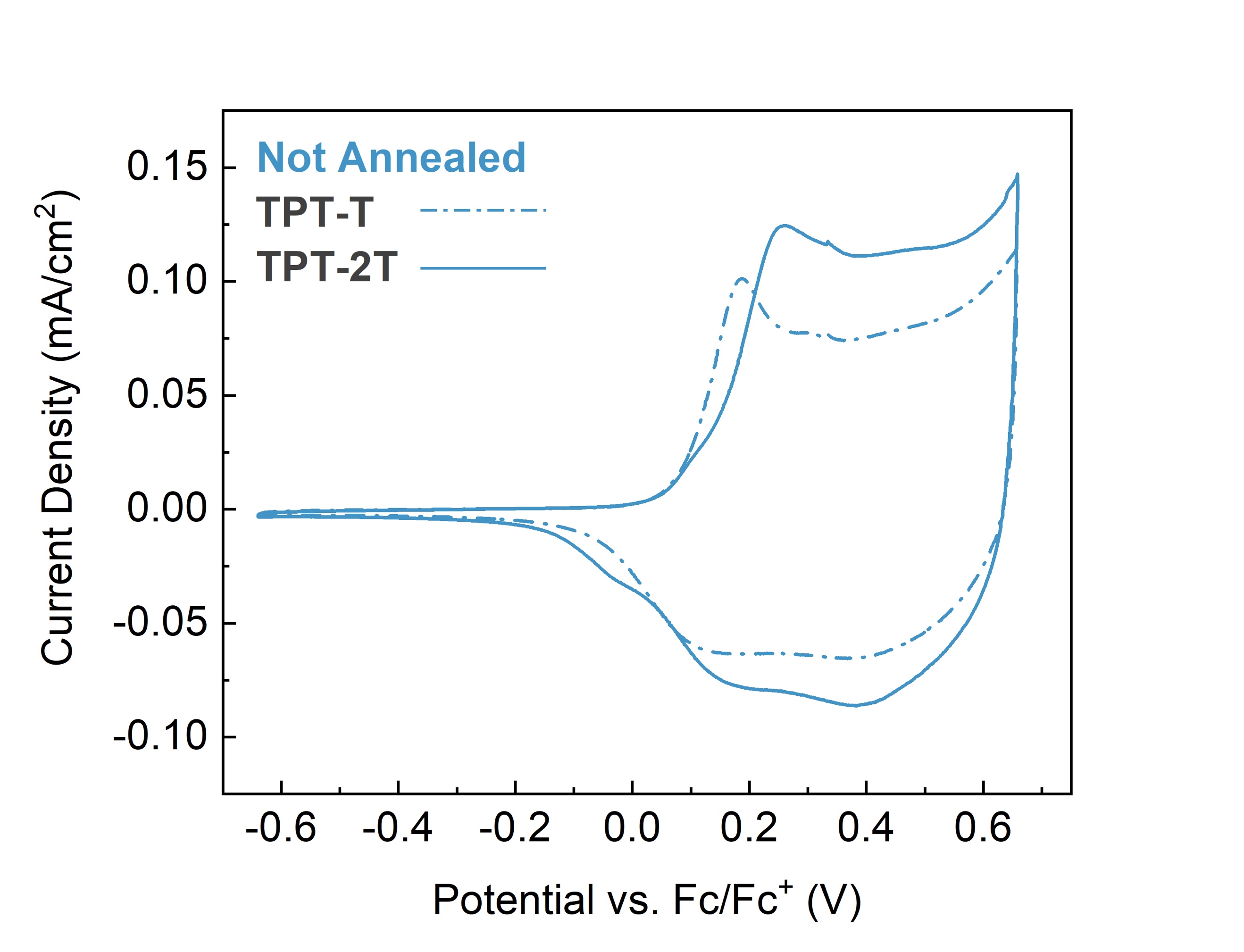

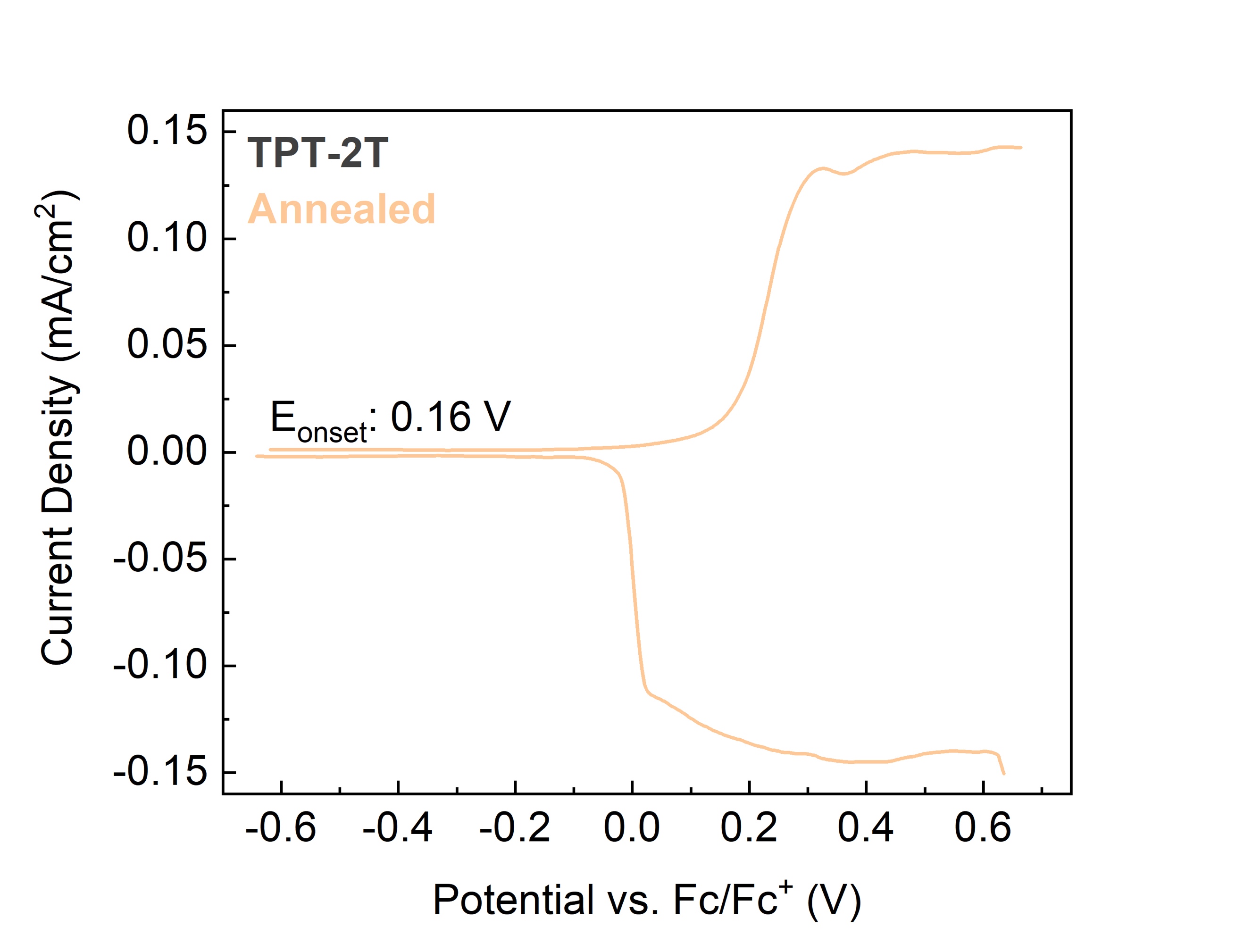

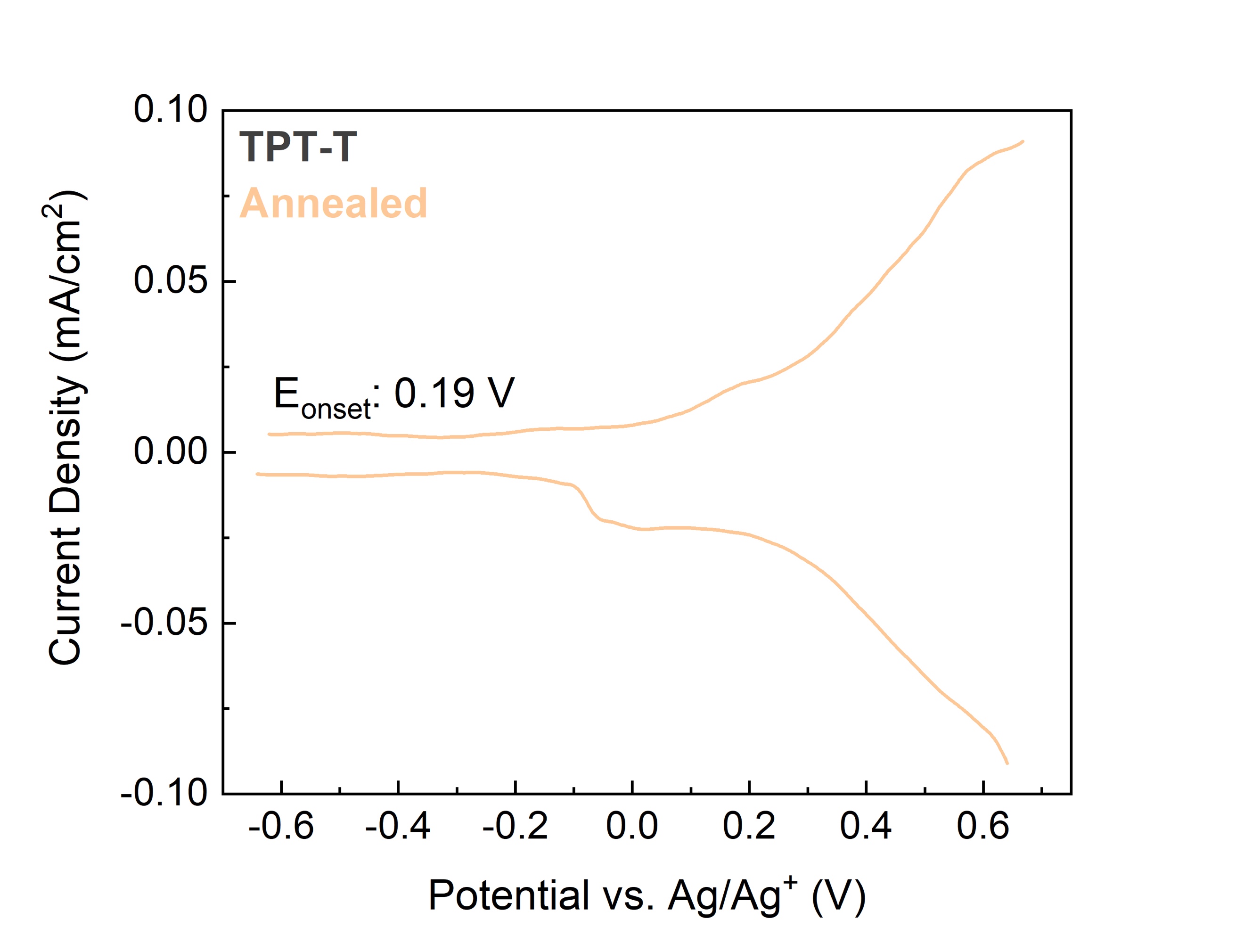


a)

b)

d)

c)

**Figure S11**. Differential pulse voltammograms of annealed a) TPT-T and b) TPT-2T films coated on ITO/glass and measured in 0.1 M TBAPF_6_/ACN. Cyclic voltammograms of c) as-cast TPT-T (dashed lines) and TPT-2T (solid lines) and of d) annealed TPT-T (dashed lines) and TPT-2T (solid lines) films on ITO/glass in 0.1 M TBAPF_6_/ACN. The scan rate used was 25 mV/s. The thermally annealed films were annealed from 20 to 250 °C under argon atmosphere at a ramp rate of 10 °C/min followed by slow cooling to room temperature. Films were cast from chloroform solutions.


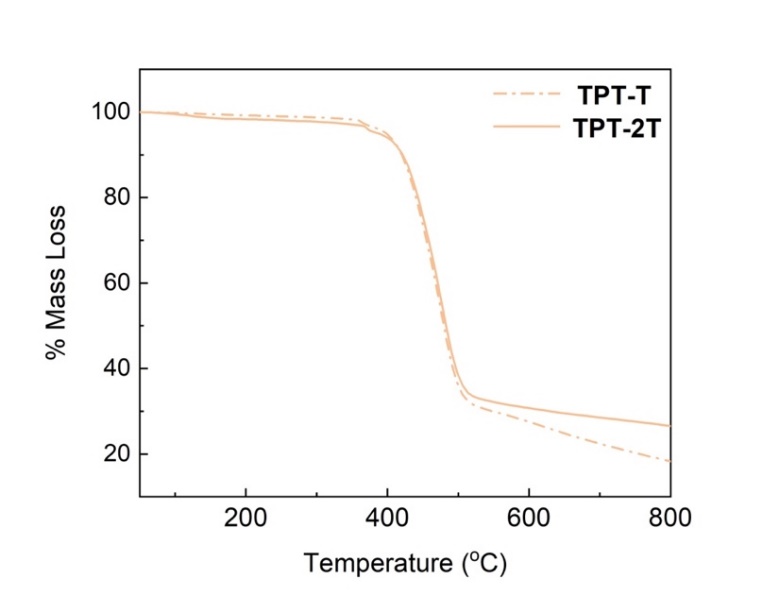


**Figure S12**. Thermogravimetric analysis of TPT-T and TPT-2T were performed on a Mettler-Toledo TGA2 by ramping the temperature from 50 °C to 800 °C at a rate of 20 °C/min under nitrogen environment. TPT-T and TPT-2T are thermally stable with an onset of thermal decomposition at 400 °C (5 % weight loss), i.e., well above the thermal transitions observed in DSC.

**Figure S13**. In situ CPOM imaging of thermal cycling of TPT-2T polymer powder.


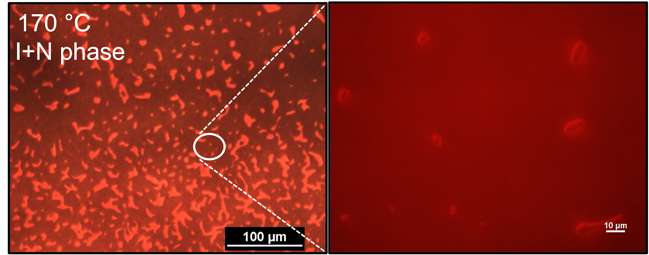


**Figure S14**. Emergence of tacoids of TPT-2T polymer powder in biphasic (isotropic + nematic) phase.


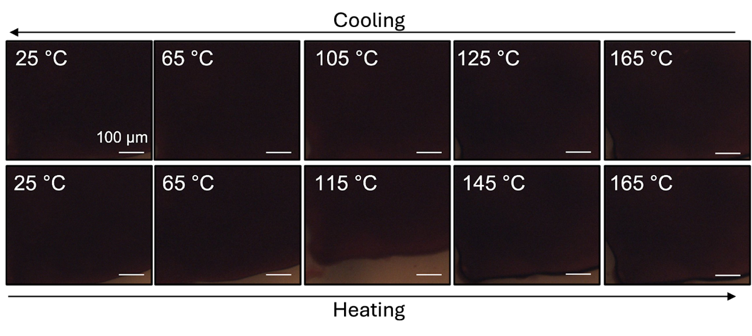


**Figure S15**. In situ CPOM imaging of TPT-T polymer powder during thermal cycling at a cooling/heating rate of 10 °C/min.

**Figure S16**. a) GIXD image of annealed TPT-2T and labeled χ angles for linecuts. b) Assessment of relative π-π crystallites population at χ = 25° and χ = 85°. The y axis represents the product of intensity I(χ) and sin(χ). c) Linecuts at χ = 25°. d) Linecuts at χ = 85°.

**Figure S17**. a) GIXD image of an annealed TPT-T and labeled χ angles for linecuts. b) Assessment of relative π-π crystallites population at χ = 5° and χ = 65°. The y axis represents the product of intensity I(χ) and sin(χ). c) Linecuts at χ = 5°. d) Linecuts at χ = 65°.

**Figure S18.** UV-vis spectra of blade coated films of a) TPT–T and b) TPT-2T films before (blue trace) and after (orange trace) annealing. The films thermally annealed from 20 to 250 °C under argon atmosphere at a ramp rate of 10 °C/min followed by slow cooling to room temperature. Films were cast from chloroform solutions. The reduced absorption intensity in annealed TPT-2T likely stems from the absorption coefficient decreasing due to the emergence of helically twisted chains, which may reduce overall absorption efficiency.^2^


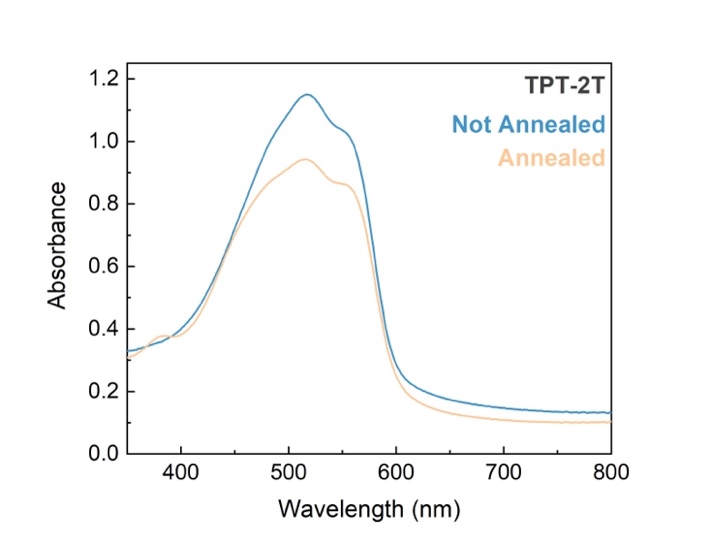

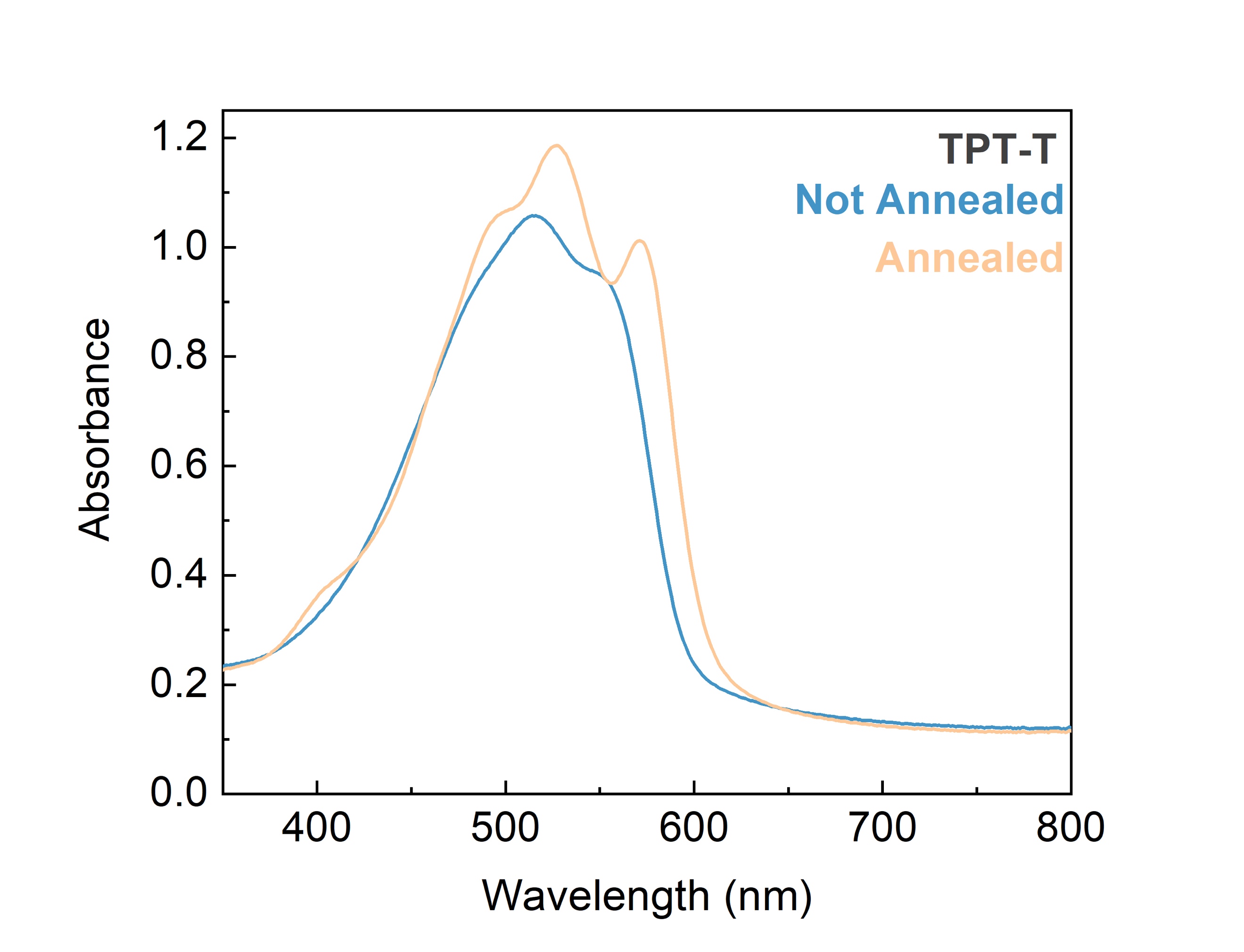


a)

b)

**Crystal-structure Proposal for TPT-T**

Multiple TPT-T conformers were constructed with varying lamellar and π-π stacking patterns by adjusting the P-T* and T*-T dihedral angles (**Figure 5**). These predesigned structures were then optimized using DFT calculations within the generalized gradient approximation (GGA) framework, employing the Perdew–Burke–Ernzerhof (PBE) functional as implemented in the Vienna Ab Initio Simulation Package (VASP). ^3–5^ For conformers with a single TPT-T monomer per repeat unit, a Monkhorst-Pack k-point grid of 2 × 3 × 13 was used, while a grid of 4 × 3 × 13 was employed for conformers with two TPT-T monomers per repeating cell. In all cases, utilized a plane-wave cutoff energy of 500 eV was considered and atomic positions were relaxed until the forces on all atoms were reduced to less than 0.01 eV/Å. van der Waals interactions were evaluated on the basis of DFT-D3 corrections. ^6^ For conformers with a density exceeding 1.0 g/cc, GIXD patterns were simulated using SimDiffraction^7^ and compared with experimentally observed data.

**Table S2**. Lattice parameters of proposed crystal structures of TPT-T along with their densities.

| TPT-T | a (Å) | b (Å) | c (Å) | α (°) | β (°) | γ (°) | Density (g/cc) |
| --- | --- | --- | --- | --- | --- | --- | --- |
| **A** | 15.70 | 27.53 | 5.27 | 66.00 | 53.13 | 100.80 | 1.06 |
| **B** | 15.74 | 20.00 | 5.35 | 59.63 | 74.19 | 99.84 | 1.10 |
| **C** | 31.63 | 25.12 | 5.22 | 58.17 | 68.52 | 102.54 | 1.02 |
| **D** | 15.66 | 21.12 | 5.25 | 59.18 | 72.71 | 95.17 | 1.05 |


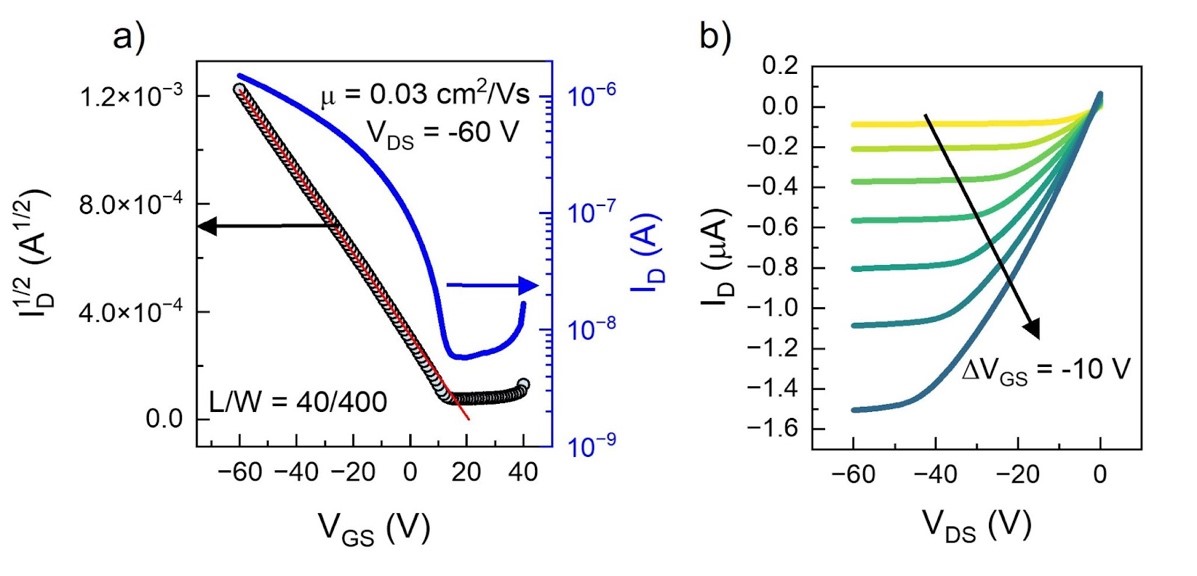


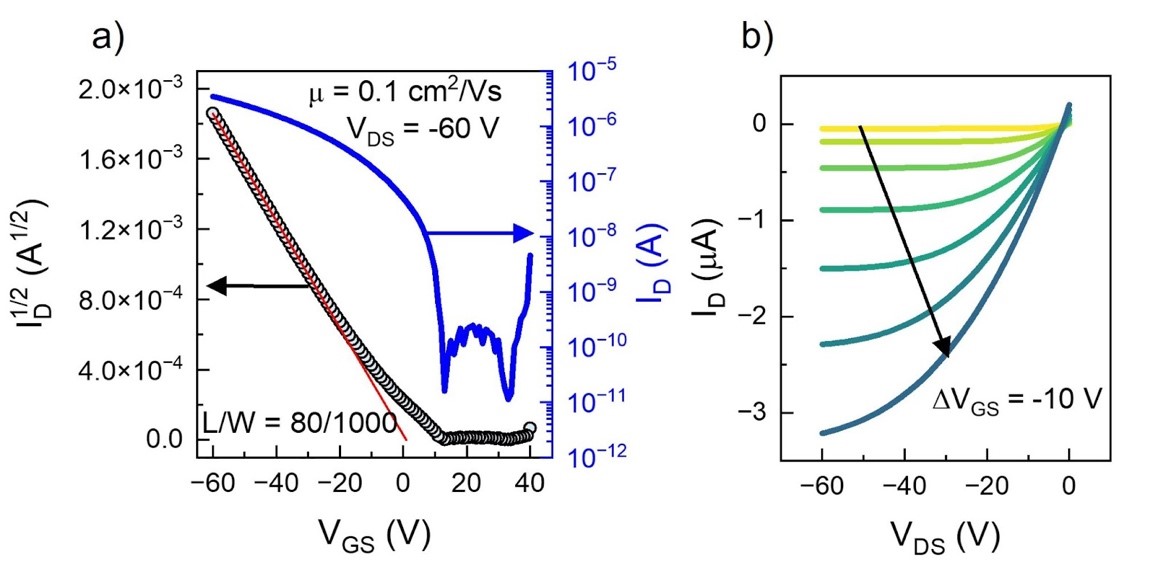
**Figure S19**. Transfer (a) and output (b) characteristics of OFETs based on as-cast TPT-T film.

**Figure S20**. Transfer (a) and output (b) characteristics of OFETs based on an annealed TPT-2T film.


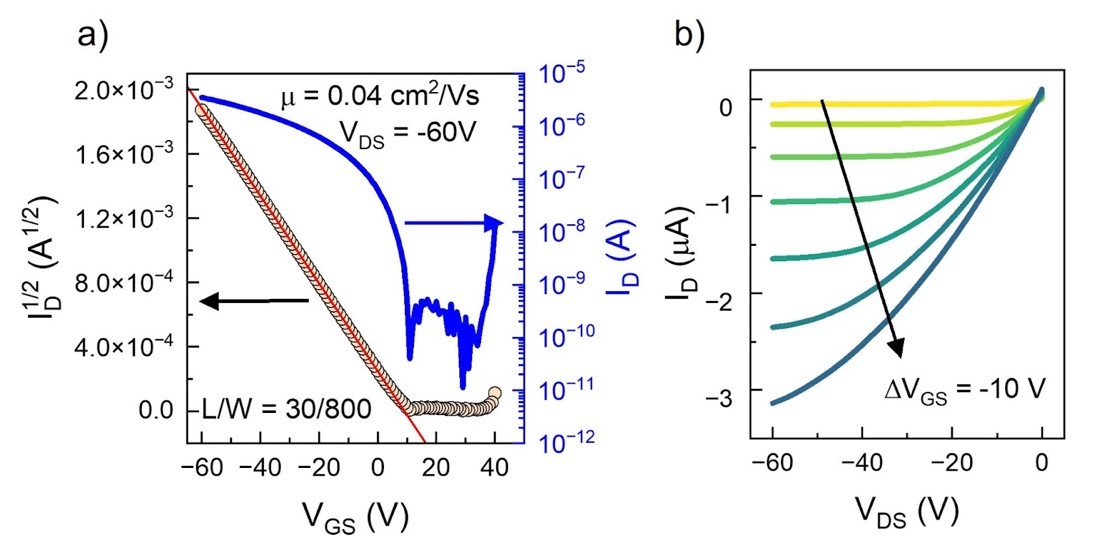
 **Figure S21**. Transfer (a) and output (b) characteristics of OFETs based on an as-cast TPT-2T film.


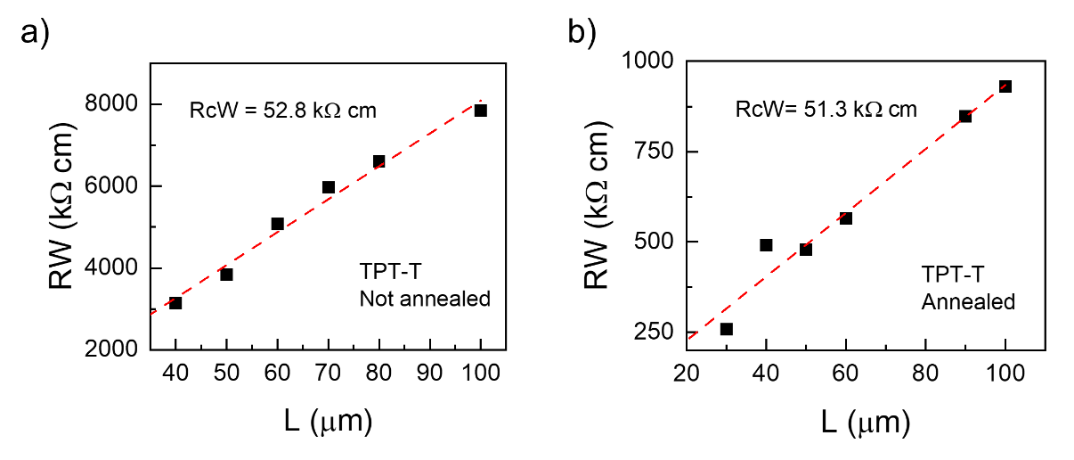


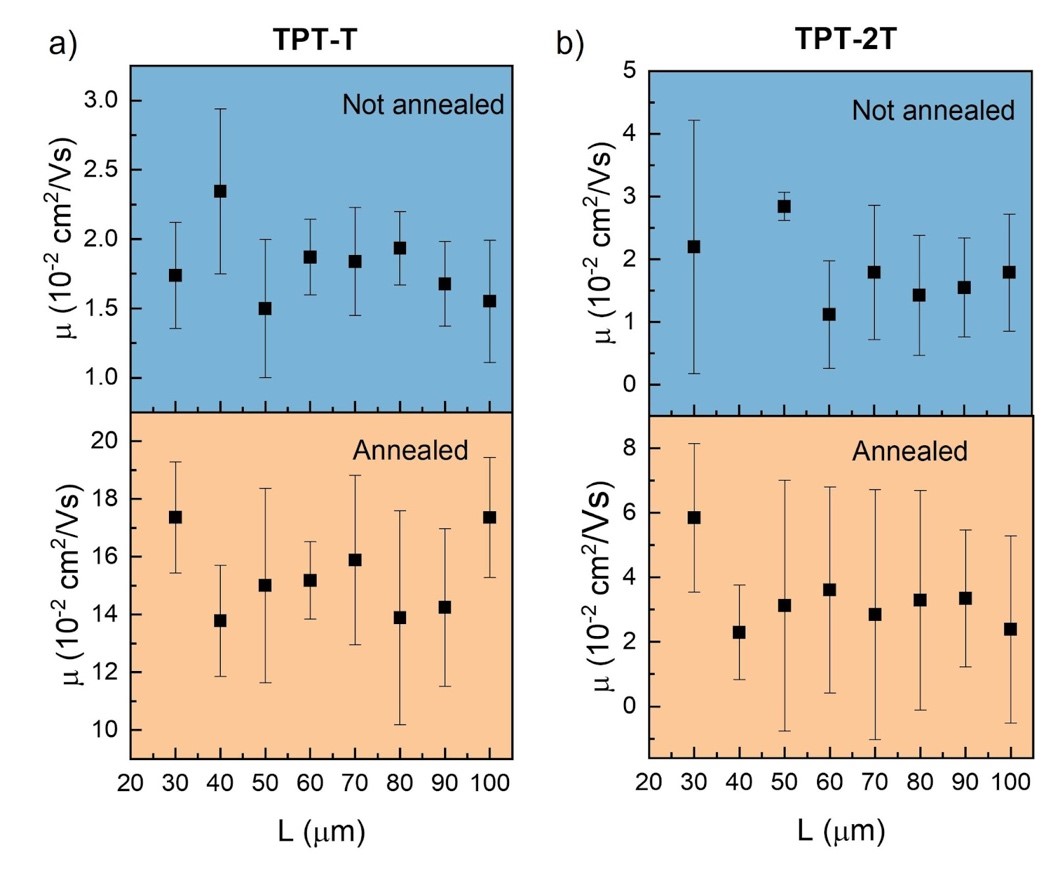
**Figure S22**. Width-normalized contact resistance of TPT-T-based OFETs: (a) before annealing and (b) after annealing.

**Figure S23**. Channel length dependence on hole mobilities of OFETs based on a) TPT-T and b) TPT-2T films.


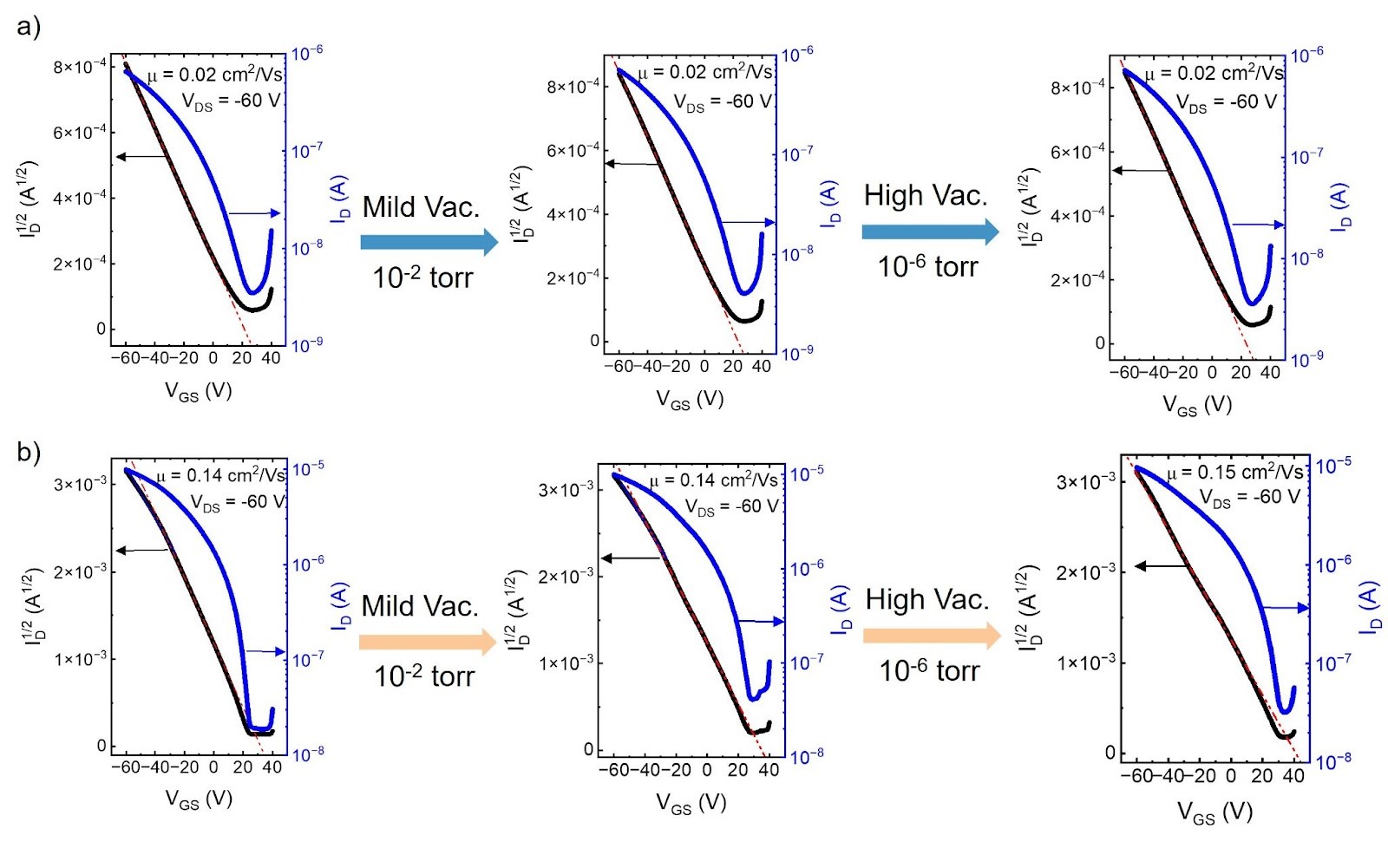


**Figure S24.** Influence of measuring environment on hole mobilities of OFETs based on a) as cast and b) annealed TPT-T films.

**References**

(1) Sabury, S.; Xu, Z.; Saiev, S.; Davies, D.; Österholm, A. M.; Rinehart, J. M.; Mirhosseini, M.; Tong, B.; Kim, S.; Correa-Baena, J.-P.; Coropceanu, V.; Jurchescu, O. D.; Brédas, J.-L.; Diao, Y.; Reynolds, J. R. Non-Covalent Planarizing Interactions Yield Highly Ordered and Thermotropic Liquid Crystalline Conjugated Polymers. *Mater. Horiz.* **2024**, *11* (14), 3352–3363. DOI:10.1039/d3mh01974h.

(2) Vezie, M. S.; Few, S.; Meager, I.; Pieridou, G.; Dörling, B.; Ashraf, R. S.; Goñi, A. R.; Bronstein, H.; McCulloch, I.; Hayes, S. C.; Campoy-Quiles, M.; Nelson, J. Exploring the Origin of High Optical Absorption in Conjugated Polymers. *Nat. Mater.* **2016**, *15* (7), 746–753. DOI:10.1038/nmat4645.

(3) Kresse, G.; Furthmüller, J. Efficiency of Ab-Initio Total Energy Calculations for Metals and Semiconductors Using a Plane-Wave Basis Set. *Comput. Mater. Sci.* **1996**, *6* (1), 15–50. DOI:10.1016/0927-0256(96)00008-0.

(4) Perdew, J. P.; Burke, K.; Ernzerhof, M. Generalized Gradient Approximation Made Simple. *Phys. Rev. Lett.* **1996**, *77* (18), 3865–3868. DOI:10.1103/PhysRevLett.77.3865.

(5) Perdew, J. P.; Burke, K.; Ernzerhof, M. Generalized Gradient Approximation Made Simple [Phys. Rev. Lett. 77, 3865 (1996)]. *Phys. Rev. Lett.* **1997**, *78* (7), 1396–1396. DOI:10.1103/PhysRevLett.78.1396.

(6) Grimme, S.; Antony, J.; Ehrlich, S.; Krieg, H. A Consistent and Accurate *Ab Initio* Parametrization of Density Functional Dispersion Correction (DFT-D) for the 94 Elements H-Pu. *J. Chem. Phys.* **2010**, *132* (15), 154104. DOI:10.1063/1.3382344.

(7) Breiby, D. W.; Bunk, O.; Andreasen, J. W.; Lemke, H. T.; Nielsen, M. M. Simulating X-Ray Diffraction of Textured Films. *J. Appl. Crystallogr.* **2008**, *41* (2), 262–271. DOI:10.1107/S0021889808001064.
